# Rap1 Activity Is Essential for Focal Adhesion and Slit Diaphragm Integrity

**DOI:** 10.3389/fcell.2022.790365

**Published:** 2022-03-18

**Authors:** Mee-Ling Maywald, Cara Picciotto, Carolin Lepa, Luisa Bertgen, Farwah Sanam Yousaf, Andrea Ricker, Jürgen Klingauf, Michael P. Krahn, Hermann Pavenstädt, Britta George

**Affiliations:** ^1^ Medizinische Klinik D, University Hospital Münster, Münster, Germany; ^2^ Institute of Medical Physics and Biophysics, Westfälische Wilhelms-University Münster, Münster, Germany; ^3^ Medizinische Klinik D, Medical Cell Biology, University Hospital Münster, Münster, Germany

**Keywords:** nephrin, podocyte, nephrocyte, Rap1, integrin β

## Abstract

Glomerular podocytes build, with their intercellular junctions, part of the kidney filter. The podocyte cell adhesion protein, nephrin, is essential for developing and maintaining slit diaphragms as functional loss in humans results in heavy proteinuria. Nephrin expression and function are also altered in many adult-onset glomerulopathies. Nephrin signals from the slit diaphragm to the actin cytoskeleton and integrin β1 at focal adhesions by recruiting Crk family proteins, which can interact with the Rap guanine nucleotide exchange factor 1 C3G. As Rap1 activity affects focal adhesion formation, we hypothesize that nephrin signals *via* Rap1 to integrin β. To address this issue, we combined *Drosophila in vivo* and mammalian cell culture experiments. We find that Rap1 is necessary for correct targeting of integrin β to focal adhesions in *Drosophila* nephrocytes, which also form slit diaphragm-like structures. In the fly, the Rap1 activity is important for signaling of the nephrin ortholog to integrin β, as well as for nephrin-dependent slit diaphragm integrity. We show by genetic interaction experiments that Rap1 functions downstream of nephrin signaling to integrin β and downstream of nephrin signaling necessary for slit diaphragm integrity. Similarly, in human podocyte culture, nephrin activation results in increased activation of Rap1. Thus, Rap1 is necessary for downstream signal transduction of nephrin to integrin β.

## Introduction

Diseases of the renal glomerulus often result in end-stage renal disease (Wiggins, 2007). In many diseases of the glomerulus, a specialized glomerular epithelial cell called podocyte plays a central role (Pavenstädt et al., 2003; Wiggins, 2007). The loss of podocytes into the urine is a pathophysiological component and a progressive factor for many glomerular pathologies (Pavenstädt et al., 2003). Podocytes are cells with a complex morphology with cellular processes that branch into foot processes (Pavenstädt et al., 2003). With their foot processes, podocytes wrap the glomerular capillaries where blood is filtered, and primary urine is generated (Scott and Quaggin, 2015). Neighboring podocyte foot processes are connected by the slit diaphragm, a specialized intercellular junction. The slit diaphragm is built by cell adhesion molecules such as nephrin and Neph1, as well as other proteins (Pavenstädt et al., 2003). Nephrin is essential for the kidney filter as mutations result in nephrotic syndrome in early childhood, which is recapitulated in nephrin knockout mice (Kestilä et al., 1998; Hamano et al., 2002). Furthermore, nephrin expression is altered in many adult-onset glomerular diseases (George and Holzman, 2012).

Nephrin is an Ig-domain family protein that functions as a transmembrane receptor in complex with Neph1 and the stomatin-family protein podocin, which anchors nephrin and Neph1 in the lipid raft membranes at the slit diaphragm (Barletta et al., 2003; Garg et al., 2007a). Among other post-translational modifications, nephrin can be phosphorylated by the tyrosine kinase Fyn, which then results in the recruitment of several signaling proteins, including the guanine nucleotide exchange factor for the small GTPase Rap1 called Rapgef1 (thereafter called C3G) (Huber et al., 2003; Verma et al., 2003; Jones et al., 2006; Verma et al., 2006; Garg et al., 2007b; Zhu et al., 2008; Jones et al., 2009; Garg et al., 2010; Zhu et al., 2010; Venkatareddy et al., 2011; George et al., 2012; George et al., 2014; Ni et al., 2016; Martin et al., 2018; Dlugos et al., 2019; Zhu et al., 2019). This initiates distinct downstream signaling events, which regulate actin cytoskeletal organization, cell survival, nephrin trafficking, slit diaphragm integrity, and integrin β1 targeting (Huber et al., 2003; Verma et al., 2003; Jones et al., 2006; Verma et al., 2006; Garg et al., 2007b; Zhu et al., 2008; Jones et al., 2009; Garg et al., 2010; Zhu et al., 2010; Venkatareddy et al., 2011; George et al., 2012; George et al., 2014; Ni et al., 2016; Martin et al., 2018; Dlugos et al., 2019; Zhu et al., 2019).

Small GTPases are versatile regulators of many cellular processes. Their action can be fine-tuned by guanine nucleotide exchange factors (GEFs) and GTPase activating proteins (GAPs). Exchange factors activate small GTPases by catalyzing the exchange of GDP by GTP while GAPs inactivate their specific GTPase by catalyzing the hydrolyzation of GTP to GDP and phosphate. The small GTPase Rap1 is ubiquitously expressed and plays important roles in controlling metabolic processes, cytoskeletal rearrangements, cell division, substratum adhesion, intercellular junction regulation, and cell motility (Lagarrigue et al., 2016; Jaskiewicz et al., 2018). In podocytes, Rap1 interacts with nephrin via the adapter protein MAGI-1, thereby regulating slit diaphragm integrity (Ni et al., 2016). Another MAGI family protein, MAGI-2, forms a complex with the Rap1 activating protein Rapgef2 to regulate Rap1 activity (Zhu et al., 2019). In mice, knockout of Rap1A and Rap1B results in disruption of slit diaphragm integrity and development of focal segmental glomerulosclerosis (FSGS)—a chronic glomerular disease with scarring, sclerotic lesions (Potla et al., 2014).

Focal adhesions are contacts between cells and the extracellular matrix (Ginsberg, 2014). Integrins are transmembrane proteins that establish these contacts (Ginsberg, 2014). α- and β-integrins heterodimerize, thereby recruiting adapter proteins, which transmit signals to the actin cytoskeleton and intercellular junctions of the cells, among others (Ginsberg, 2014). In podocytes, integrin β1 is one of the major β-integrins (Sachs and Sonnenberg, 2013). Integrin function is essential to podocytes as knockout of integrin β1 in mice results in early proteinuria as a sign of defective slit diaphragm development (Pozzi et al., 2009). These mice show a progressive podocyte loss presumably due to adhesion defects (Pozzi et al., 2009). Nephrin transduces signals to integrin β1 at focal adhesions by recruiting the Rap1 activating GEF C3G, which results in activation and correct targeting of integrin β1 (Dlugos et al., 2019).

While Rap1 plays a role in nephrin signaling that mediates slit diaphragm integrity (Ni et al., 2016), the role of Rap1 for nephrin signal transduction to integrin β at focal adhesions is not defined yet. Employing the *Drosophila* nephrocyte model, we show that Rap1 is necessary for mediating signals that correctly target integrin β to focal adhesions. Furthermore, Rap1 activity is relevant for nephrin signaling to regulate slit diaphragm integrity. By genetic interaction experiments, we show that Rap1 functions downstream of nephrin to regulate integrin β function at focal adhesions in nephrocytes. Likewise, we show that nephrin activation in podocyte culture results in increased activation of Rap1.

## Methods

### Fly Husbandry and Genetics

For the transgenic RNAi and overexpression studies, the *UAS-Gal4* system was employed. Flies were kept at 25°C or 29°C on standard food. RNAi stocks were obtained from the Vienna Drosophila Resource Center (Vienna, Austria): control RNAi targeting *orco* (100825), *rap1* RNAi (20761), *rap1* RNAi (110757) and *magi* RNAi (41735). UAS-Rap1^V12^ (constitutively active), UAS-Rap1^N17^ (dominant-negative), and UAS-Rap1 (wild-type) were a gift from Ulrike Gaul (LMU, Munich, Germany). The overexpression control UAS RFP (30556) was obtained from the Bloomington Stock Center (Bloomington, United States). UAS Sns and Sns-Gal4 driver lines were provided from Tobias Huber (UKE, Hamburg, Germany). For the rescue experiments, driver lines were recombined with the specific RNAi fly lines.

### Generation of Transgenic Flies

The pENTR™⁄DTOPO® Kit (ThermoFisher) was used for cloning of h-Rap1B V12, h-Rap1B N17, and h-Rap1B WT into pENTR (human Rap1B DNA was provided from Andreas Püschel, WestfälischeWilhelms-Universität Münster, Germany). Cloning into the destination vector was performed using the Gateway technology (Invitrogen). UAS-h-Rap1B^V12^, UAS-h-Rap1B^N17^, and UAS-h-Rap1B (wild-type) transgenic fly lines were generated using *ΦC31*-mediated germline transformation using landing sites *attP40* and *attP2*.

### Immunofluorescence Analysis

Wandering third instar larvae were dissected and the garland cell nephrocytes fixed in 4% paraformaldehyde (PFA) for 15 min. After washing in PBS-T (PBS + 0.1% Triton), nephrocytes were incubated in the primary antibody at 4° overnight, followed by washes in PBS-T. The secondary antibody incubation lasted for 2.5 h at room temperature. After washes with PBS-T, they were mounted in Mowiol. Anti-Sns [custom generated (Dlugos et al., 2019)], anti-Pyd (PYD2) and anti-integrin β (CF.6G11) (all from Developmental Studies Hybridoma Bank, United States) were used as primary antibodies. Anti-Sns antibodies were diluted 1:100, anti-Pyd antibodies 1:20, anti-integrin β antibodies 1:100 in PBS-T. Goat-anti-mouse Alexa^488^, goat-anti-rabbit Alexa^594^, and DAPI (all from Invitrogen) were used as secondary antibodies and diluted 1:1000 in 10% goat serum in PBS-T.

### Confocal Microscopy

Tangential und surface section images of the nephrocytes were obtained using a confocal microscope (Leica SP8). 1.5× zoom was set for the tangential section and 4× zoom for the surface section. For imaging, the integrated module LIGHTNING was applied. Image processing was done by LasX and ImageJ Software.

### Transmission Electron Microscopy

Wandering third instar larvae were dissected, and garland cell nephrocytes were fixed in 2% glutaraldehyde in Sørensen buffer overnight. Next, nephrocytes were washed with Sørensen buffer, osmium tetroxide 1% in Sørensen buffer was applied for 1 h, and samples were again washed with Sørensen buffer. Samples were then dehydrated in an ascending alcohol series and infiltrated with epon using a series of mixtures of epon and the intermedium propylene oxide and pure epon. After embedding in epon and polymerization at 60°C for 36 h, samples were cut in ultra-thin slices of 60 nm and contrasted with uranyl acetate for 20 min and lead citrate for 90 s. Images were taken with a transmission electron microscope (Phillips CM10 equipped with TVIPS CAM F416).

### Statistical Analysis

Quantification of slit diaphragms visualized across 1 μm was performed manually (LasX Software, EM measure). The Mann–Whitney *U* test was used to determine the statistical significance between two interventions. Slit diaphragms were quantified in 14 nephrocytes from 20 different animals for each of three independent crosses per genotype. Slit diaphragms were counted perpendicularly across 5 µm cell membrane in five different areas of each cell. To detect integrin β localization upon downregulation of rap1, ROIs were selected at the cell cortex and the cytosol close to the nucleus. The integrin reorganization was then calculated as previously described (Bayraktar et al., 2020): ROI at cell cortex + background area/ROI at cytosol + background area. Slit diaphragms were counted per µm basement membrane for 10 nephrocytes, each of 20–30 animals employing transmission electron microscopy images. Basement membrane length was quantified using ImageJ.

### Cell Culture

Human immortalized podocytes were cultured as previously described (Dlugos et al., 2019). Stable podocyte cell lines allowing doxycycline-dependent expression of CD16-CD7-Nephrin Cytoplasmic Domain (NCD) or CD16-CD7-HA (pInducer-21-puro-CD16-CD7-NCD or pInducer-21-puro-CD16-CD7-HA) were generated by lentiviral gene transfer as previously described (Dlugos et al., 2019).

### Nephrin Activation Assay

Activation of nephrin was performed as previously described (Dlugos et al., 2019). In short, either CD16-CD7-NCD or CD16-CD7-HA (as control) stable cell lines cultured on 10 cm dishes were cooled down to 4°C for 5 min, incubated with an anti-CD16 antibody (#555404, BD Pharming, 4 µl per ml medium) on ice for 30 min, and then further incubated with an anti-IgG antibody (#ab6708, Abcam, 3 µl per ml medium) for another 5 min at 37°C. The first incubation step allows the anti-CD16 antibody to bind to the CD16 domain of the chimeric proteins and leads to a first clustering of the proteins. The second incubation step allows the binding of the anti-IgG antibody to the bound anti-CD16 antibodies for an enhancement of clusters, which initiates recruitment of Src kinases and consecutive phosphorylation of the intracellular nephrin domain (Verma et al., 2003; Jones et al., 2006; Verma et al., 2006).

### Active Rap1 Detection

After nephrin activation by clustering, cells were directly cooled down on ice, media was removed, and cells were washed with ice-cold PBS. Cells were then lysed in 500 µl ice-cold IP buffer (20 mM Tris-HCl, pH 7.4; 20 mM NaCl; 1 mM EDTA; 50 mM NaF; 15 mM Na_4_P_2_O_7_; 1% (v/v) Triton X-100) containing protease inhibitor (cOmplete™ protease inhibitor cocktail, Roche Diagnostics) and phosphatase inhibitors (phosphatase inhibitor cocktail 2 and phosphatase inhibitor cocktail 3, Sigma Aldrich) and manually scratched from the plates. Further mechanical lysis was achieved by dragging the lysates through a 26, G needle. Lysates were kept on ice for 30 min, whereby they were vortexed every 3 to 5 min and were then centrifuged for 15 min at 14.000 × g and 4°C. The supernatant was transferred into a new reaction tube containing Sepharose G beads (GE Healthcare), which were washed three times for 5 min rotating in 1 ml IP buffer. 5% of the lysate was kept aside as input. 1 µl of active Rap1-GTP antibody (#26912, NewEast Biosciences) was added to 30 µl of beads slurry and lysate. Immunoprecipitation was performed rotating at 4°C for 1 h. Afterward, beads were washed three times at 4°C, rotating with 1 ml IP buffer each time, containing protease and phosphatase inhibitors. Following the washing steps 2× Laemmli was added to the beads, and the samples were boiled at 95°C for 5 min. Immunoblotting was performed as previously described (Dlugos et al., 2019). Primary antibodies were diluted 1:1000 in 5% bovine serum albumin in TBST. The following antibodies were used: Rap1A/B (#2399S, Cell Signaling Technologies), nephrin (#BP5030, OriGene), p-nephrin (#ab80298, Abcam), HA (#11867423001, Roche), β-tubulin (#T8328, Sigma Aldrich).

## Results

### Rap1 Is Essential for Correct Targeting of Integrin β in Nephrocytes

We recently showed that nephrin activation results in the activation of integrin β1 in podocytes (Dlugos et al., 2019). C3G appears to play a role in this pathway (Dlugos et al., 2019). C3G is an activator of the small GTPase Rap1 (Radha et al., 2011). However, the role of Rap1 in nephrin signaling to focal adhesions in podocytes is unclear. To analyze the function of Rap1 in nephrin signaling to integrin β, we combined the use of two model systems: podocyte culture and the *in vivo Drosophila* nephrocyte model. The nephrocyte expresses a slit diaphragm-like structure based on sticks and stones (Sns, ortholog of nephrin) and Kin-of-irre (Kirre, ortholog of Neph1) (Weavers et al., 2009; Zhuang et al., 2009). Like in podocytes, the nephrocyte slit diaphragm functions as a size and charge-selective barrier (Zhang et al., 2013a). In analogy to podocytes, nephrocytes express the cytoskeletal adapters Cindr (ortholog of CD2AP), Myoblast city (Mbc), Nck ortholog), and Mec-2 (ortholog of podocin) (Weavers et al., 2009; Zhuang et al., 2009). Thus, this model is currently often employed as an *in vivo* model for analyzing the slit diaphragm in a genetically tractable system (Zhang et al., 2013a; Zhang et al., 2013b; Fu et al., 2017; Hermle et al., 2017; Hochapfel et al., 2017).

**FIGURE 1:**
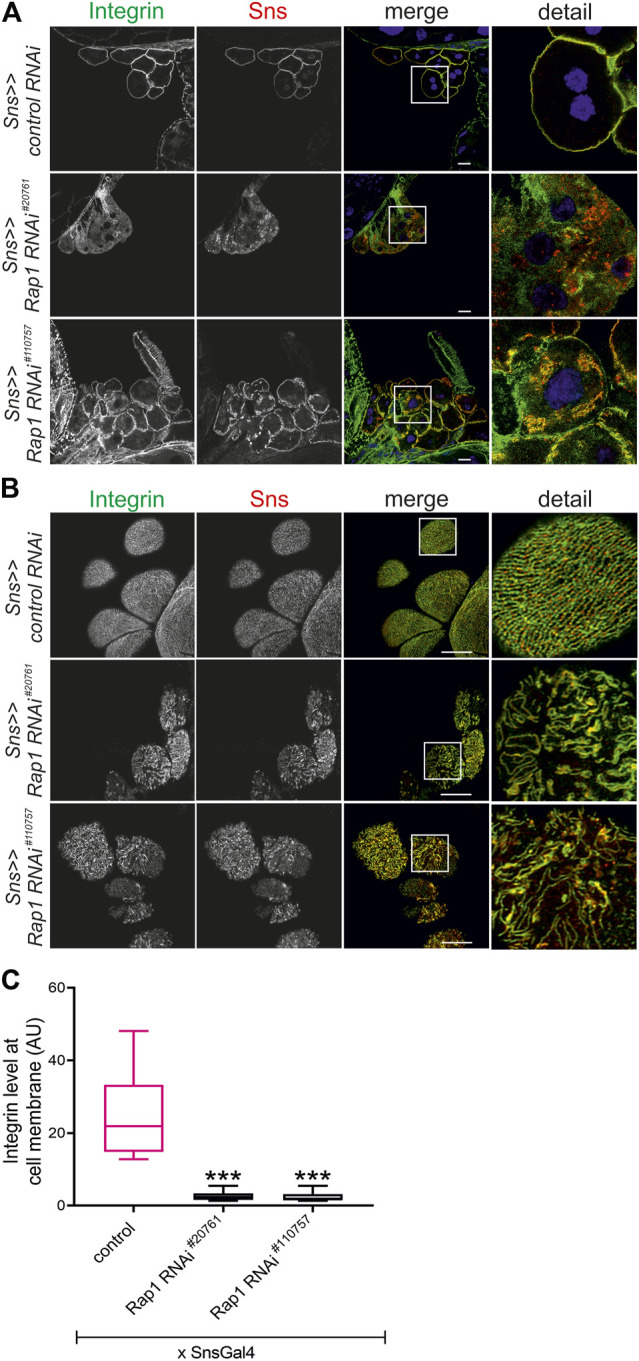
Knockdown of *rap1* results in altered targeting of integrin β. **(A)** Immunofluorescence analysis of tangential sections **(A)** or surface sections **(B)** of *control* (*sns >> control RNAi*) or *rap1* knockdown nephrocytes (*sns>>rap1 RNAi*) is shown. Knockdown in nephrocytes was accomplished by employing *sns-GAL4* and two different RNAi hairpins (#20761 and #110757). Nephrocytes of wandering third instar larvae were dissected, and immunofluorescence analysis was performed with antibodies specific for integrin β (green) and Sns (red). Merged images and higher magnifications of the marked area (detail) are shown. Scale bars in **(A,B)**: 10 µm, *n* = 3. **(C)** The intensity of integrin fluorescence at the cell membrane compared to the cytosol was evaluated for all genotypes shown in **(A)** and presented as a box plot with minimum and maximum in arbitrary units (AU). The control is marked in pink. *** indicates a *p*-value *<* 0.001.

**FIGURE 2:**
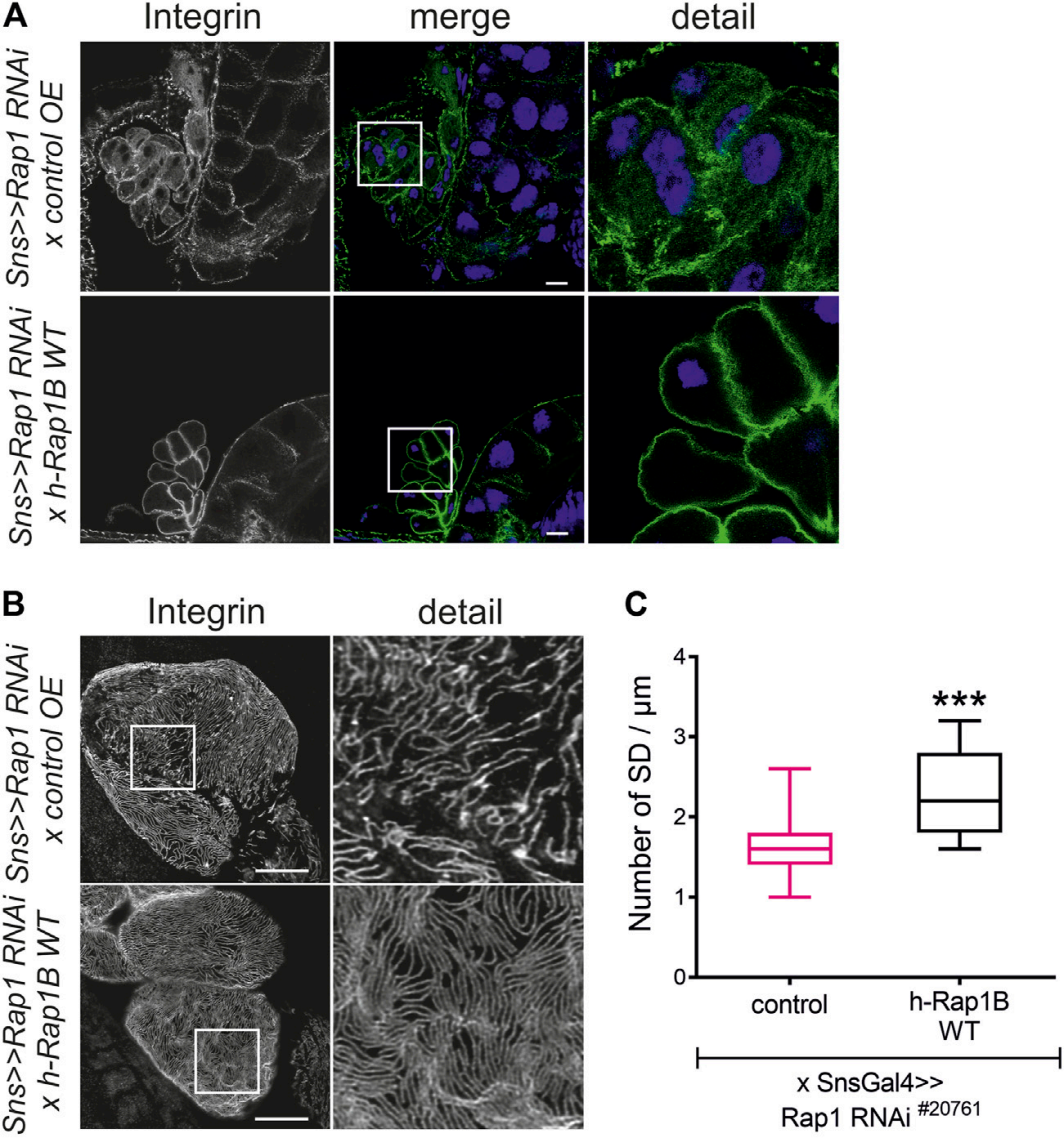
Human Rap1B can rescue *rap1* loss of function-induced mislocalization of integrin β. Immunofluorescence analysis of tangential **(A)** or surface sections **(B)** of *rap1* knockdown nephrocytes (*sns>>rap1 RNAi*) with a genetic element for overexpression of human Rap1B (*UAS h-Rap1B WT*) or a control transgene (*control OE*) is shown. Knockdown of *rap1* (#20761) in nephrocytes was accomplished by employing *sns-GAL4*. Wandering third instar larvae were dissected, and immunofluorescence analysis was performed with antibodies specific for integrin β. Merged images and higher magnifications of the marked area (detail) are shown. Scale bars in **(A,B)**: 10 µm, *n* = 3. **(C)** Statistical evaluation of the number of slit diaphragms (SD) per µm nephrocyte surface area of the genotypes shown in **(A,B)** depicted as a box plot with minimum and maximum. The control is marked in pink. ***p < 0.001.

To test whether Rap1 is essential for targeting of integrin β, we downregulated endogenous *rap1* exclusively in nephrocytes employing the *UAS-Gal4* system with a nephrocyte-specific driver (*sns-Gal4*) and two independent dsRNA lines targeting *rap1*. The localization of myospheroid, the *Drosophila* ortholog of integrin β, was determined in nephrocytes of third instar larvae using confocal microscopy. In tangential sections, integrin β exclusively accumulated at the cell cortex in control nephrocytes while nephrocytes with knockdown of *rap1* showed diffuse cytoplasmic staining of integrin β with some aggregates (Figure 1A). When imaging the surface of nephrocytes, integrin β presented in a typical fingerprint-like pattern in control nephrocytes (Figure 1B), whereas its localization was severely altered with diffuse targeting of integrin β in *rap1* knockdown nephrocytes (Figure 1B). Integrin accumulation at the plasma membrane was quantified in tangential sections (Figure 1C) (Bayraktar et al., 2020). To confirm that integrin β mistargeting is a specific effect of *rap1* downregulation, we performed rescue experiments of the *rap1* knockdown by expressing human Rap1B, which is not targeted by the dsRNA specific to the *Drosophila rap1* gene. Nephrocytes expressing a control element [*UAS-RFP*, called *control OE* (overexpression)] instead of human Rap1B on *rap1* knockdown background were prepared, and immunofluorescence analysis was performed with antibodies specific for integrin β (genotype: *sns-Gal4*, *UAS rap1RNAi* ^*20761*^/*UAS RFP* or *sns-Gal4*, *UAS rap1RNAi* ^*110757*^/*UAS RFP*). Analysis of tangential (Figure 2A, Supplementary Figure S1A) and surface sections (Figure 2B, Supplementary Figure S1B) of nephrocytes confirmed that downregulation of *rap1* resulted in diffuse targeting of integrin β. Upon knockdown of *rap1* and concomitant expression of *UAS-h-Rap1B*, the phenotype induced by silencing of *rap1* was partly rescued. In these animals, integrin β was localized correctly at the plasma membrane (Figure 2A, Supplementary Figure S1A) and was found in the fingerprint-like pattern in surface sections (Figure 2B, Supplementary Figure S1B) (genotypes: *sns-Gal4*, *rap1RNAi* ^*20761*^/*UAS h-Rap1B; UAS h-Rap1B/Tm6b* or *sns-Gal4*, *rap1RNAi* ^*110757*^/*UAS h-Rap1B; UAS h-Rap1B/Tm6b).* We quantified the number of foot processes per 1 µm of the basement membrane, which confirmed the qualitative results (Figure 2C, Supplementary Figure S1C).

### Rap1 Activity Is Necessary for Targeting of Integrin β

**FIGURE 3:**
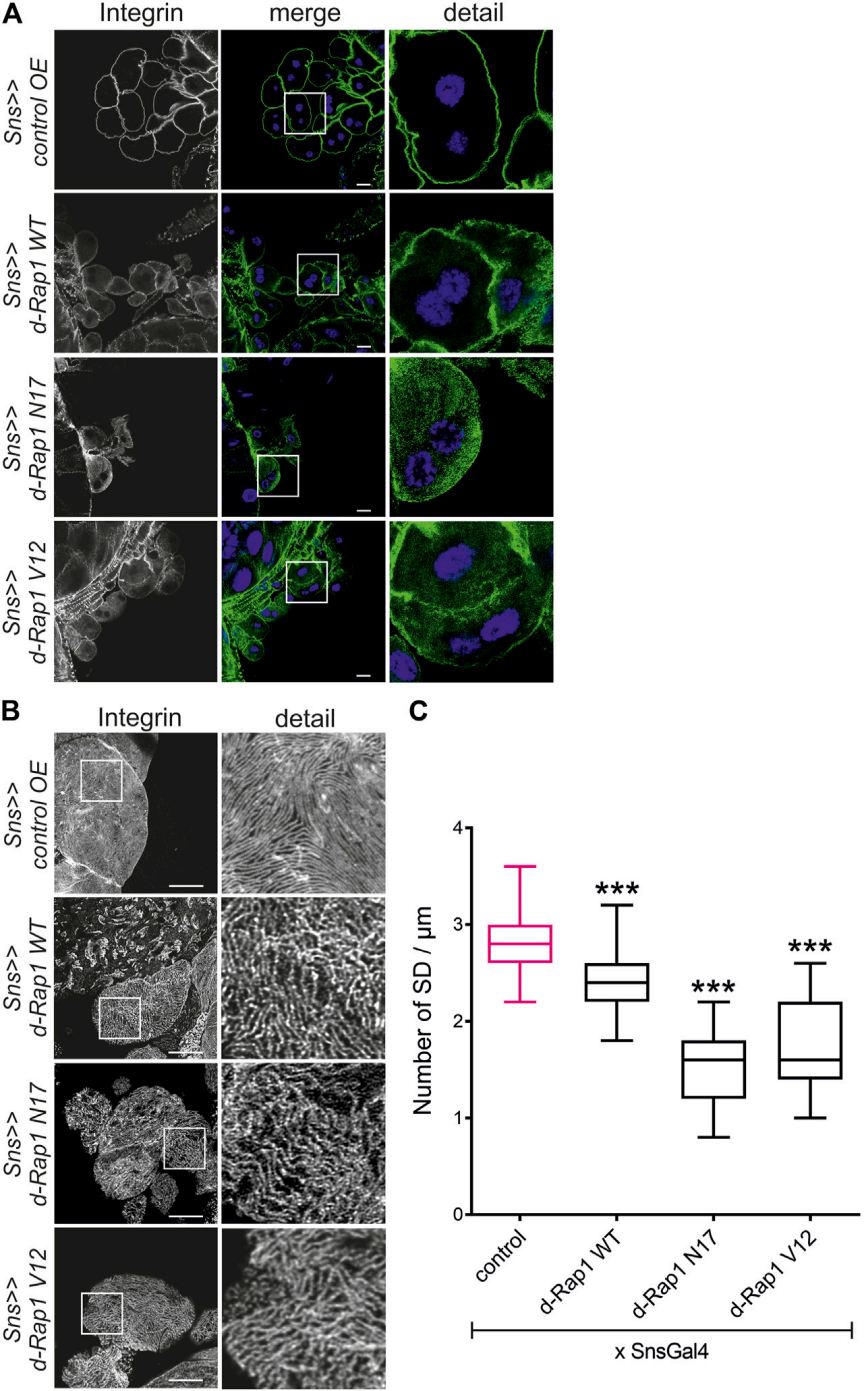
Imbalance in *rap1* activity results in altered targeting of integrin β. Immunofluorescence analysis of tangential **(A)** or surface sections **(B)** of control nephrocytes (*sns>>control OE*), nephrocytes with overexpression of *Drosophila rap1* (*sns>>d-rap1 WT*), of *Drosophila* dominant-negative *rap1* (*sns>>d-rap1 N17*), or of *Drosophila* constitutively active *rap1* (*sns>>d-rap1 V12*). Knockdown in nephrocytes was accomplished by employing *sns-GAL4*. Wandering third instar larvae were dissected, and immunofluorescence analysis was performed with antibodies specific for integrin β. Merged images and higher magnifications of the marked area (detail) are shown. Scale bars in **(A,B)**: 10 µm, *n* = 3. **(C)** Statistical evaluation of the number of slit diaphragms (SD) per µm nephrocyte surface area of the genotypes shown in **(A,B)** depicted as a box plot with minimum and maximum. The control is marked in pink. ***p < 0.001.

**FIGURE 4:**
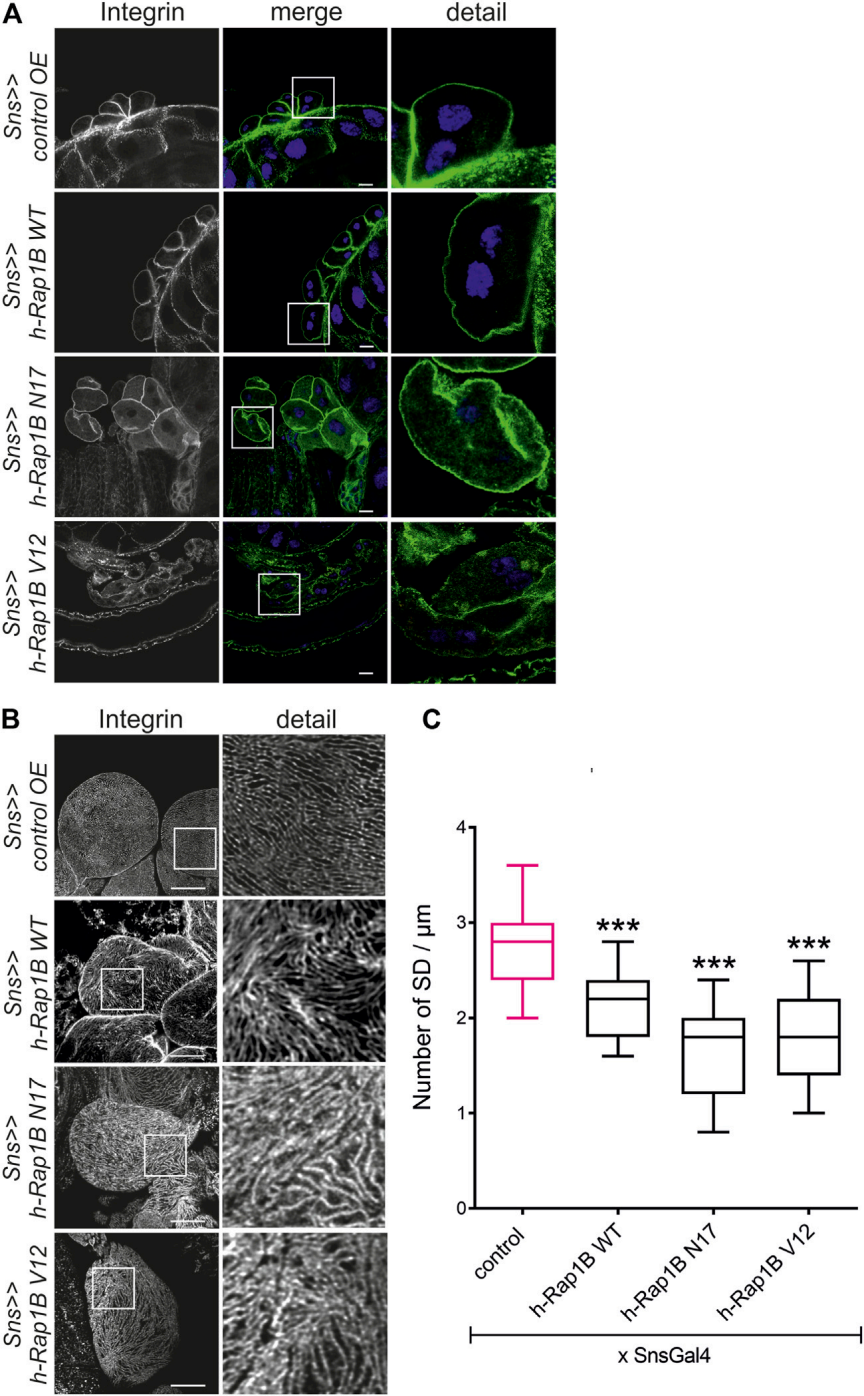
Misexpression of human Rap1B results in altered targeting of integrin β. Immunofluorescence analysis of tangential **(A)** or surface sections **(B)** of control nephrocytes (*sns>>control OE*), nephrocytes with overexpression of wild-type human Rap1B (*sns>>h-Rap1B WT*), of human dominant-negative Rap1B (*sns>>h-Rap1B N17*), or of human constitutively active Rap1B (*sns>>h-Rap1B V12*). Knockdown in nephrocytes was accomplished by employing *sns-GAL4*. Wandering third instar larvae were dissected, and immunofluorescence analysis was performed with antibodies specific for integrin β. Merged images and higher magnifications of the marked area (detail) are shown. Scale bars in **(A,B)**: 10 µm, *n* = 3. **(C)** Statistical evaluation of the number of slit diaphragms (SD) per µm nephrocyte surface area of the genotypes shown in **(A,B)** depicted as a box plot with minimum and maximum. The control is marked in pink. ***p < 0.001.

The activation state of small GTPases is tightly regulated by activating and inactivating factors (Lagarrigue et al., 2016; Jaskiewicz et al., 2018). To deduce whether the activity state of Rap1 is important for correct targeting of integrin β, we expressed wild-type *Drosophila* Rap1, dominant-negative Rap1^N17^ with amino acid substitution of serine to asparagine at position 17, constitutively active Rap1^V12^ with amino acid substitution of glycine to valine at position 12 or RFP as a control in wild-type nephrocytes. Compared to the control (genotype: *sns-Gal4/UAS RFP*), overexpression of *rap1* resulted in moderate mistargeting of integrin β, which was more severe when overexpressing either constitutively active or dominant-negative Rap1 (genotypes: *sns-Gal4/UAS d-rap1* or *sns-Gal4/UAS d-rap1N17* or *sns-Gal4; d-rap1V12*) (Figures 3A–C). To test whether ectopic expression of human Rap1B variants exerts a similar phenotype to their *Drosophila* orthologs, we expressed human h-Rap1B, h-Rap1B^V12^, h-Rap1B^N17^, or RFP as a control on the wild-typic background (genotypes: *sns-Gal4/UAS RFP* or *sns-Gal4/UAS h-Rap1B; UAS h-Rap1B* or *sns-Gal4/UAS h-Rap1B* ^N17^ *; UAS h-Rap1B* ^N17^ *or sns-Gal4/UAS h-Rap1B* ^V12^ *; UAS h-Rap1B* ^V12^). Tangential sections (Figure 4A) or surface sections (Figures 4B,C) showed that misexpression of all three h-Rap1B variants resulted in mistargeting of integrin β, demonstrating that the function of *rap1* in focal adhesion integrity appears to be conserved.

### Rap1 Activity Is Essential for Nephrocyte Slit Diaphragm Integrity

**FIGURE 5:**
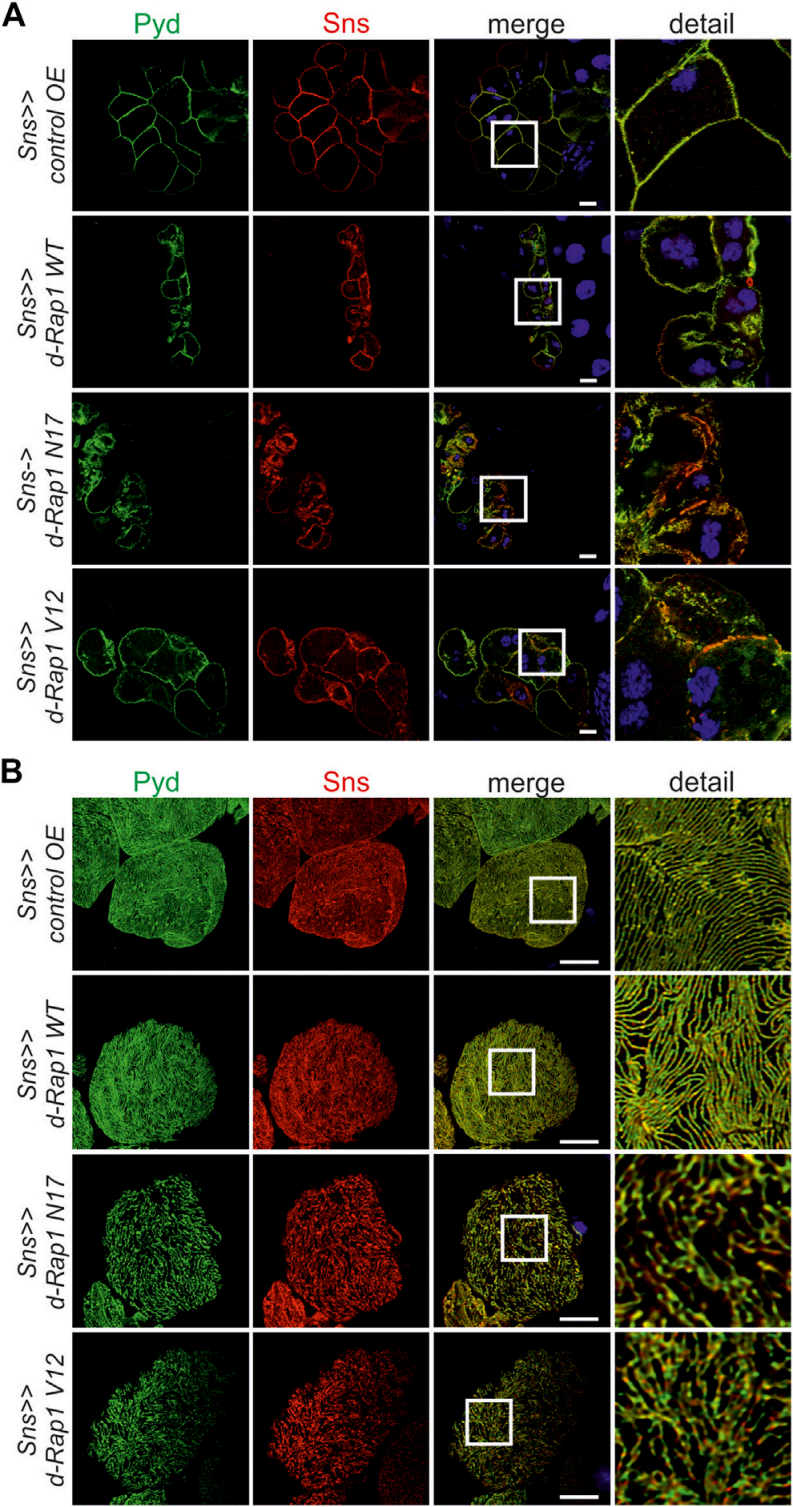
Imbalance of *rap1* activity leads to mistargeting of slit diaphragm proteins Pyd and Sns. Immunofluorescence analysis of tangential **(A)** or surface sections **(B)** of control nephrocytes (*sns>>control OE*), nephrocytes with overexpression of *Drosophila* wild-type *rap1* (*sns>>d-rap1 WT*), of *Drosophila* dominant-negative *rap1* (*sns>>d-rap1 N17*), or of *Drosophila* constitutively active *rap1* (*sns>>d-rap1 V12*). Knockdown in nephrocytes was accomplished by employing *sns-GAL4*. Wandering third instar larvae were dissected, and immunofluorescence analysis was performed with antibodies specific for Pyd (zonula occludens-1 (ZO-1) ortholog) and Sns (nephrin ortholog) as slit diaphragm markers. Merged images and higher magnifications of the marked area are shown in the right columns. Scale bars in **(A,B)**: 10 µm, *n* = 3.

**FIGURE 6:**
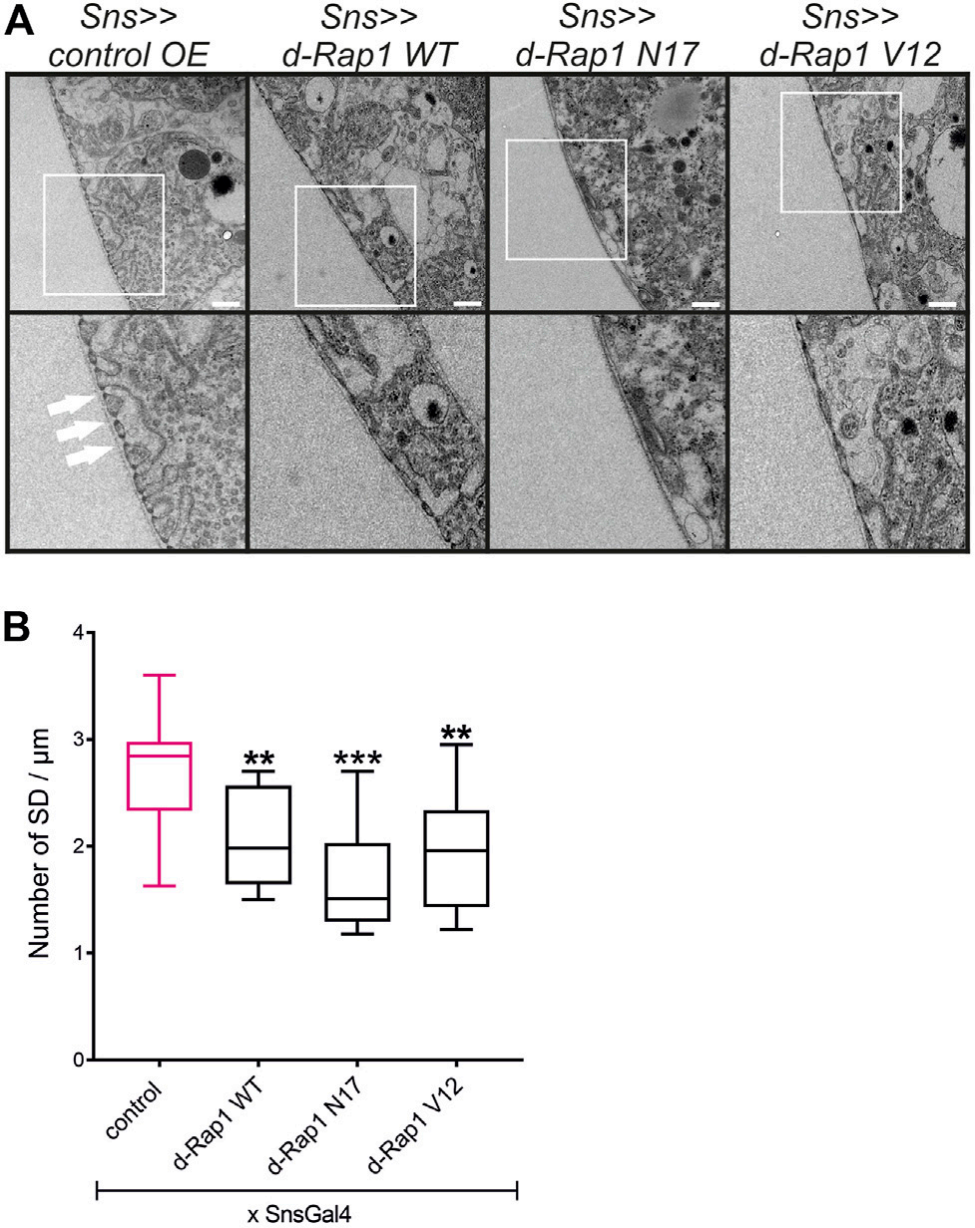
Imbalance of *d-rap1* activity leads to loss of slit diaphragms and lacunae. **(A)** TEM analysis of dissected control nephrocytes (*sns>>control OE*), nephrocytes with overexpression of *Drosophila* wild-type *rap1* (*sns>>d-rap1 WT*), of *Drosophila* dominant-negative *rap1* (*sns>>d-rap1 N17*), or of *Drosophila* constitutively active *rap1* (*sns>>d-rap1 V12*). Knockdown in nephrocytes was accomplished by employing *sns-GAL4*. White arrows exemplarily indicate slit diaphragms. Scale bars: upper panel 500 nm, lower panel zoom compared to the upper panel 2×. *n* = 3. **(B)** Statistical evaluation of the number of slit diaphragms (SD) per µm basement membrane of the genotypes shown in **(A)** depicted as a box plot with minimum and maximum. The control is marked in pink. **p < 0.01, ***p < 0.001.

In mice, Rap1 is essential for slit diaphragm integrity (Potla et al., 2014). In the *Drosophila* nephrocyte model, the role of Rap1 in slit diaphragm integrity has not yet been characterized. Knockdown of *rap1* by two different *dsRNA* in nephrocytes *in vivo* employing *sns-Gal4* resulted in incorrect targeting of the slit diaphragm proteins Sns and Pyd (the fly ortholog of Zonula occludens (ZO-1) in tangential and surface sections (Supplementary Figures S2A,B). Next, we analyzed whether the expression of h-Rap1B can rescue the *rap1* knockdown phenotype of slit diaphragm protein mistargeting. This showed that expression of h-Rap1B could rescue the *rap1* knockdown-related phenotype of Sns and Pyd mistargeting induced by two independent *dsRNA* (Supplementary Figures S3A,B, S4A,B). Thus, the *Drosophila* nephrocyte model recapitulates the phenotypes induced by Rap1 loss of function in mice. We then utilized the nephrocyte model to characterize the role of Rap1 for slit diaphragm integrity further. It is presently not clear whether a balanced Rap1 activity is crucial for slit diaphragm integrity in mammalian podocytes *in vivo* or *Drosophila* nephrocytes. In contrast to the overexpression control (RFP), nephrocyte-specific overexpression of *rap1*, *rap1* ^*V12*^, and *rap1* ^*N17*^ on the wild-typic background resulted in mistargeting of slit diaphragm proteins, Sns and Pyd. Similar to targeting of integrin β to focal adhesions, targeting of Sns and Pyd to the slit diaphragm was disturbed by expressing wild-type *rap1* or the constitutively active or dominant-negative forms of *rap1* (Figures 5A,B). To analyze whether this correlates with the breakdown of slit diaphragms, we performed transmission electron microscopy (TEM) analysis. Nephrocyte-specific overexpression of either wild-type, dominant-negative or constitutively active *rap1* led to a reduction of slit diaphragms per µm basement membrane (Figures 6A,B). In analogy, misexpression of wild-type human Rap1B, constitutively active h-Rap1B^V12^ or dominant-negative h-Rap1B^N17^ on wild-typic background resulted in misexpression of Sns and Pyd and breakdown of slit diaphragms (Supplementary Figures S5A,B, S6). Thus, slit diaphragm integrity appears to be regulated by Rap1 activity.

### Rap1 Functions Downstream of Nephrin During Signaling to Integrin β and Is Essential for Nephrin-Mediated Slit Diaphragm Integrity

**FIGURE 7:**
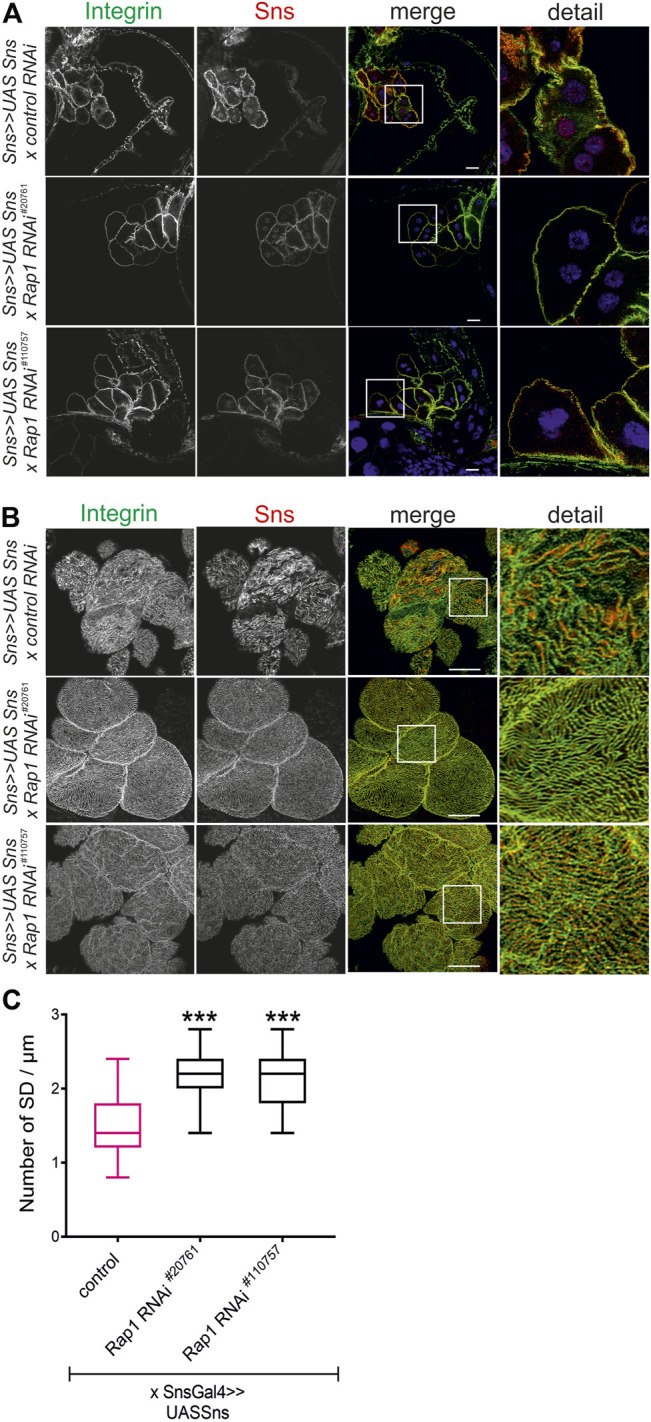
Rap1 functions downstream of nephrin in signaling to integrin β. Immunofluorescence analysis of tangential **(A)** or surface sections **(B)** of nephrocytes overexpressing *sns* (*sns>>UAS Sns x control RNAi*) or nephrocytes with overexpression of sns and knockdown of *rap1* (*sns>>UAS Sns x Rap1 RNAi* ^*20761*^ or *sns>>UAS Sns x Rap1 RNAi* ^*110757*^). Knockdown in nephrocytes was accomplished by employing *sns-GAL4*. Wandering third instar larvae were dissected, and immunofluorescence analysis was performed with antibodies specific for integrin β (green) and Sns (red). Merged images and higher magnifications of the marked area are shown. Scale bars **(A,B)**: 10 µm, *n* = 3. **(C)** Statistical evaluation of the number of slit diaphragms (SD) per µm nephrocyte surface area of the genotypes shown in **(A,B)** depicted as a box plot with minimum and maximum. The control is marked in pink. ***p < 0.001.

It is well known that *Drosophila* is excellently suited to genetically dissect complex signaling cascades (Simon et al., 1991; Fortini et al., 1992; Brunner et al., 1994). The ability to suppress a phenotype caused by overexpression of a given gene (gain-of-function phenotype) by suppressing another gene is evidence that the two genes interact (Simon et al., 1991; Fortini et al., 1992; Brunner et al., 1994). We employed this technique to test whether the nephrin ortholog *sns* genetically interacts with *rap1.* Flies overexpressing *sns* in nephrocytes were crossed with flies with nephrocyte-specific knockdown o*f rap1* (*sns-Gal4*, *UAS-sns*; *UAS-d-rap1RNAi* ^*110757*^ or *sns-Gal4*, *UAS-sns*; *UAS-d-rap1 RNAi* ^*20761*^) and compared to flies with overexpression of *sns* and a control *UAS-dsRNA* element (*sns-Gal4*, *UAS-sns*; *UAS-control RNAi*). While flies overexpressing *sns* in a background of a control *UAS-dsRNA* element showed mistargeting of integrin β in nephrocytes, flies that overexpress *sns* in the background of *rap1* suppression showed a normal integrin β localization in nephrocytes (Figures 7A–C). Importantly, two independent UAS-dsRNA elements tested could rescue integrin β targeting, implying that Rap1 functions downstream of Sns in signaling to integrin β at focal adhesions. Downregulation of *rap1* was also able to partly rescue Sns and Pyd targeting on an *sns* gain-of-function genetic background (Supplementary Figures S7A,B). Thus, Rap1 also functions downstream of Sns during signaling that regulates slit diaphragm morphology.

**FIGURE 8:**
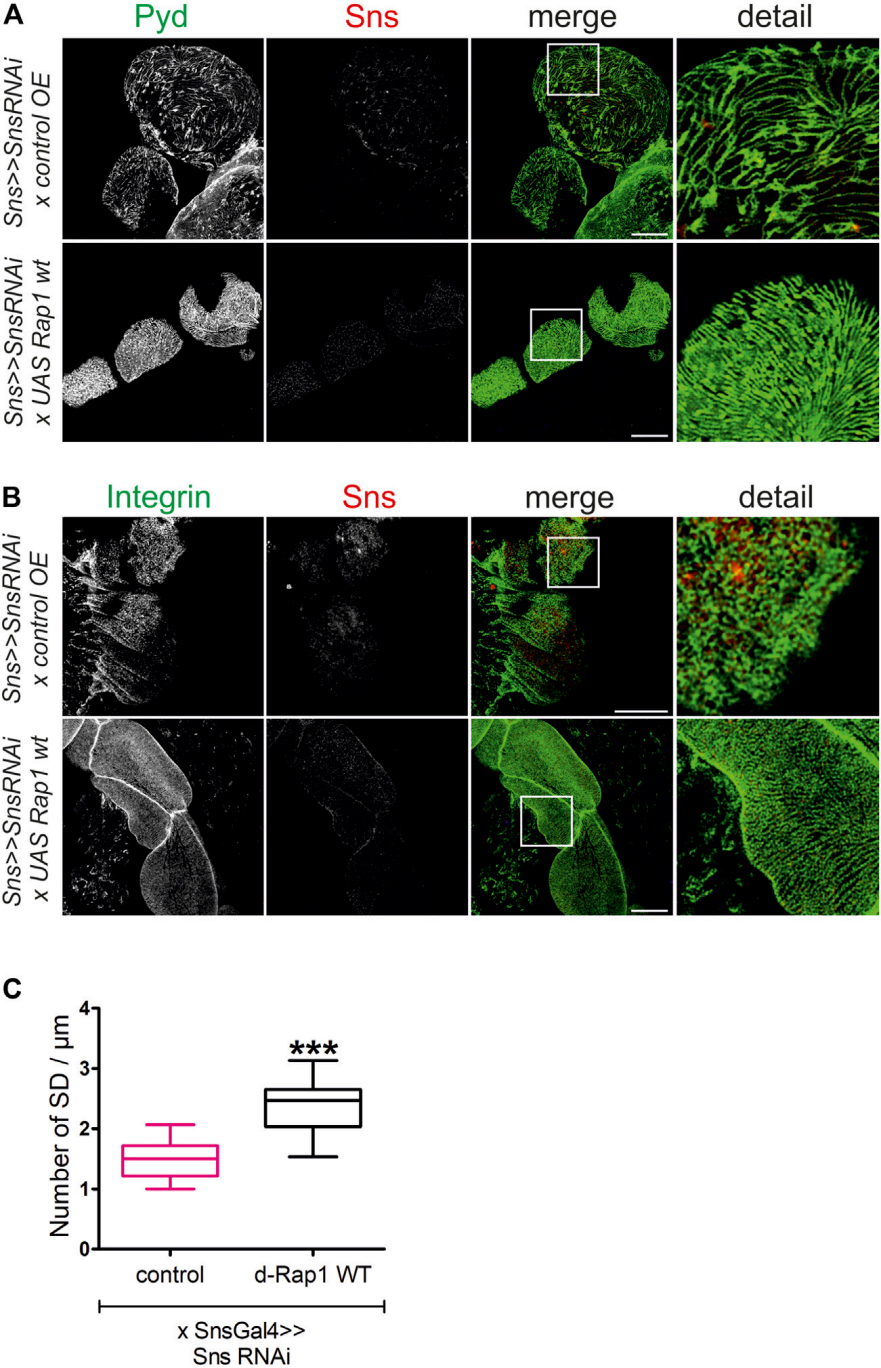
*Rap1* overexpression can rescue the loss of slit diaphragms and integrin targeting induced by *sns* downregulation. Immunofluorescence analysis of surface sections of nephrocytes with knockdown of *sns* and overexpression of a control element or overexpression of *Drosophila rap1* (*sns>>UAS snsRNAi x UAS RFP* or *sns>>UAS snsRNAi x UASdRap1*). Knockdown in nephrocytes was accomplished by employing *sns-GAL4*. Wandering third instar larvae were dissected, and immunofluorescence analysis was performed with antibodies specific for Pyd (green) and Sns (red) **(A)** or integrin β (green) and Sns (red) **(B)**. Merged images and higher magnifications of the marked area are shown. Scale bars **(A,B)**: 10 µm, *n* = 3. **(C)** Statistical evaluation of the number of slit diaphragms (SD) per µm nephrocyte surface area of the genotypes shown in **(A)** depicted as a box plot with minimum and maximum. The control is marked in pink. ***p < 0.001.

Slit diaphragm integrity and integrin targeting were also rescued when overexpressing *d-rap1* on the *sns* knockdown background (*sns-Gal4>>sns RNAi x UAS-d-rap1*, control: *sns-Gal4>>sns RNAi x UAS-control*) (Figures 8A–C). This confirms that Rap1 fulfills functions downstream of Sns.

In mammalian podocytes, Rap1 activity is also regulated via the scaffold proteins, MAGI-1 and MAGI-2. *Drosophila* has only one MAGI ortholog. To test whether dMAGI is essential for slit diaphragm integrity in nephrocytes, we performed a knockdown of the MAGI ortholog by RNAi in nephrocytes (*sns-Gal4*). Immunofluorescence analysis of *magi* knockdown nephrocytes showed a slightly reduced number of slit diaphragms when stained for Sns and Pyd to visualize slit diaphragms (Supplementary Figure S8). Thus, MAGI is also necessary for an intact slit diaphragm in nephrocytes.

### Nephrin Activation Results in Increased Active Rap1 in Podocytes

**FIGURE 9:**
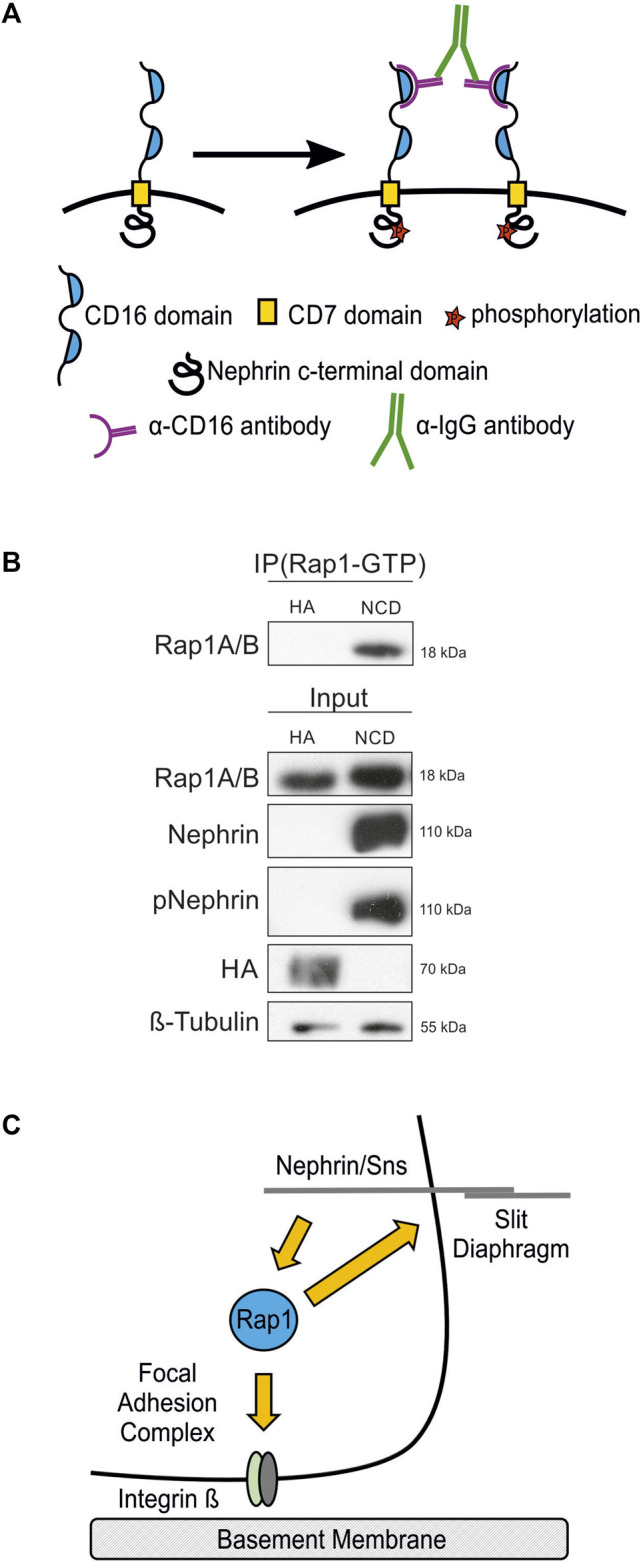
Nephrin activation results in increased active Rap1 in human podocytes. **(A)** A nephrin clustering assay was performed by adding anti-CD16 antibody and secondary anti-IgG antibody to the media of cultured podocytes, which induces clustering of nephrin proteins followed by recruitment of Src kinases and consecutive phosphorylation of nephrin on tyrosine residues. **(B)** Immunoblot with antibodies specific for Rap1A/B following immunoprecipitation (IP Rap1-GTP) with an antibody specific for active Rap1A/B of podocyte lysates. Employed were human podocyte lines that inducibly express either chimeric CD16-CD7-nephrin cytoplasmic domain (NCD) or CD16-CD7-HA (HA) as a control following doxycycline treatment followed by clustering of nephrin. Immunoblots of these lysates were also performed with antibodies specific for Rap1A/B, nephrin, phospho-nephrin (pNephrin), HA, and β-tubulin (Input), *n* = 3. **(C)** Schematic of the hypothesis that the small GTPase Rap1 functions downstream of nephrin in signaling to integrin β at focal adhesions and in signaling at the slit diaphragm.

To confirm that nephrin activation results in signaling that leads to activation of Rap1 in mammalian podocytes, we employed a podocyte culture system where nephrin signal transduction can be activated (Figure 9A) (Jones et al., 2006; Verma et al., 2006; George et al., 2012; George et al., 2014). Cultured human podocytes that inducibly express a chimeric nephrin protein by doxycycline (dox) treatment consisting of a CD16 extracellular domain, a CD7 transmembrane domain, and the cytoplasmic domain of nephrin (CD16-CD7-NCD). Incubation with anti-CD16 antibody induces clustering of chimeric nephrin, initiating recruitment of Src kinases to the nephrin intracellular domain and consecutive phosphorylation on activating tyrosine residues known to bind to cytoskeletal adapter proteins such as Nck, Crk1/2, CrkL, phospho-inositol-3-kinase (pi3k), and other signaling intermediaries (Jones et al., 2006; Verma et al., 2006; George et al., 2012; George et al., 2014). As controls, podocytes inducibly expressing CD16-CD7-HA, where the cytoplasmic domain of nephrin is replaced by HA-tag, were used. Podocyte lines were treated with dox to induce the expression of either CD16-CD7-NCD or CD16-CD7-HA followed by incubation with antibodies specific for CD16 and secondary IgG to trigger clustering. Podocyte lysates were then subjected to immunoprecipitation employing an antibody specific for GTP-bound, active Rap1A/B followed by immunoblotting with an antibody recognizing total Rap1A/B. Furthermore, immunoblots were performed with antibodies specific for nephrin, phospho-nephrin (pNephrin), HA, total Rap1A/B, and β-tubulin to control loading. This showed that the amount of the active form of Rap1-GTP was increased following activation of nephrin (Figure 9B).

## Discussion

By employing the *Drosophila* nephrocyte model, we showed that Rap1 is necessary for typical integrin localization in nephrocytes. Rap1 activity is essential for nephrin signaling to integrin β and nephrin signaling to regulate slit diaphragm integrity. We show by genetic interaction experiments that Rap1 functions downstream in nephrin signaling to integrin β at focal adhesions. In podocyte culture, nephrin activation results in increased activation of Rap1. Thus, the GTPase Rap1 is a novel intermediary in the signaling pathway from nephrin at the slit diaphragm to integrin β at focal adhesions (Figure 9C).

*Drosophila* nephrocytes emerged as a genetically tractable model to dissect slit diaphragm biology and nephrin signal transduction as nephrocytes exhibit slit diaphragms where, similar to mammals, the principal junction proteins and their cytoskeletal adaptors are present (Weavers et al., 2009; Zhuang et al., 2009; Dlugos et al., 2019). The short generation time of *Drosophila* makes it feasible to identify novel mediators in signaling pathways. In a previous study, we identified the small GTPase Rap1 to be necessary for slit diaphragm function and protein uptake in nephrocytes (Dlugos et al., 2019). *Drosophila* genetics make it possible to dissect whether genes act in the same pathway by genetic interaction experiments (Simon et al., 1991; Fortini et al., 1992; Brunner et al., 1994). By increasing nephrin expression and simultaneous downregulation of the *rap1* expression level, we showed that Rap1 acts downstream of nephrin signal transduction to integrin β and in nephrin signaling necessary for slit diaphragm integrity. Dismantling signaling cascades diminutively is essential to discover potential novel drug targets for glomerular disease therapy.

In other cell models, the role of Rap1 in governing the inside-out activation of integrin dimers that consist of a β1, β2, or β3 subunit is well-established (Bos et al., 2003; Caron, 2003; Dustin et al., 2004; Bos, 2005). In blood cells, Rap1 regulates integrin activation *via* the Rap1-GTP-interacting adapter molecule (RIAM) and consecutive activation of integrin by talin (Lagarrigue et al., 2016). Conversely, Rap1 does not appear to play a role in signaling from integrin to adhesion proteins at intercellular junctions (Balzac et al., 2005). Thus, the role of Rap1 in integrin regulation and function is currently judged to be unidirectional (Balzac et al., 2005). This is interesting, as integrin ligation indeed transduces signals that result in nephrin phosphorylation at the slit diaphragm (Verma et al., 2016), but mediators of this are not yet identified. Previously, we showed that the Rap1 activator C3G acts downstream of nephrin in the signaling pathway to integrin β (Dlugos et al., 2019). Now, we find that Rap1 regulates signaling of nephrin at the intercellular junction in nephrocytes that modulates the targeting of integrin β at focal adhesions clarifying inside-out signal transduction of integrin β in nephrocytes and podocytes.

Rap1 also plays an important role in regulating intercellular junctions (Retta et al., 2006). In endothelial cells, Rap1 activity controls endothelial barrier function by tightening VE-cadherin-based cell–cell adhesion (Cullere et al., 2005; Fukuhara et al., 2005). In contrast to the unilateral function of Rap1 in integrin activation regulation, its role at intercellular junctions appears to be bidirectional. Rap1 is also a target of E-cadherin in outside-in signaling following junction disassembly and E-cadherin endocytosis in endothelial cells (Balzac et al., 2005). In podocytes, nephrin interacts with the adapter protein MAGI-1, thereby recruiting Rap1 (Ni et al., 2016). Rap1 activity is modulated by the Rap1 activating factor Rapgef2 (Zhu et al., 2019). In mice, knockout of Rap1A and Rap1B results in disruption of slit diaphragm integrity and development of focal segmental glomerulosclerosis (FSGS) (Potla et al., 2014). Mutations in the gene encoding MAGI-2 in humans result in nephrotic syndrome, a glomerular pathology where the breakdown of the podocyte intercellular junction and consecutive filtration barrier dysfunction result in loss of protein into the urine (Ashraf et al., 2018). The chronic nephrotic syndrome often leads to chronic glomerular disease. We now show that the Rap1 activity balance is central for an intact slit diaphragm. Both increased and decreased Rap1 activities result in a phenotype of an effaced slit diaphragm. This implies that Rap1 activity is tightly regulated in the nephrin signaling pathway that regulates intercellular junction integrity and the pathway from nephrin to integrin β that governs focal adhesion composition.

We observe a similar phenotype of Sns or Rap1 gain and loss of function. This may indicate that Sns and Rap1 functions are tightly regulated by the cell, and physiological protein levels and activity are necessary for nephrocytes or podocytes. It is not uncommon that signaling cascades are dysregulated by either excess or lack of a signaling component (Reichardt et al., 2000; Feng, 2012; Toker and Chin, 2014; Wang et al., 2014). Many proteins function in multi-protein complexes. Stoichiometric changes in protein complex composition may result in dysfunction of the respective protein complex independent of the cause being excess or lack of one protein component. This could also be the case for Sns and Rap1. In contrast, we cannot rule out that overexpression of either Sns or Rap1 may cause a dominant-negative effect and thus exhibit similar phenotypes as the respective knockdowns. Furthermore, it needs to be noted that, by employing a dominant-negative or constitutively active form of Rap1, we analyzed the impact of very high or low levels of Rap1 activity on focal adhesion and slit diaphragm integrity. To conclude that Rap1 activity has to be tightly balanced in podocytes and nephrocytes to mediate focal adhesion and slit diaphragm integrity, more modest changes in Rap1 activity need to be assayed.

As MAGI is involved in nephrin-dependent Rap1 regulation and slit diaphragm integrity in mammalian podocytes, it is not surprising that dMAGI is also necessary for intact slit diaphragms in nephrocytes. The nephrocyte model can thus be employed to further characterize the role of MAGI in nephrin signaling in future studies. It will be very interesting to distinguish whether MAGI and C3G act in the same pathway down-stream of Sns or whether MAGI and C3G fulfil functions in separate pathways.

Podocyte loss is a central progression factor of chronic glomerular disease (Wiggins, 2007). Mechanisms that regulate podocyte detachment are not well understood. We now dissect a pathway from nephrin *via* Rap1 that regulates integrin β targeting and potentially podocyte adhesion. It will be interesting to further characterize this signaling pathway and its role in different glomerular diseases as this may uncover interesting targets to modify podocyte loss in glomerular disease.

## Data Availability

The original contributions presented in the study are included in the Supplementary Material, further inquiries can be directed to the corresponding author.
