# Supplementary material for: Rap1 Activity Is Essential for Focal Adhesion and Slit Diaphragm Integrity
Source: Front Cell Dev Biol. 2022 Mar 18;10:790365. doi: 10.3389/fcell.2022.790365 (PMC8972170; doi:10.3389/fcell.2022.790365)
Supplement: Supplementary file 1 [file DataSheet1.PDF]

Supplementary Material

1 Supplementary Figures

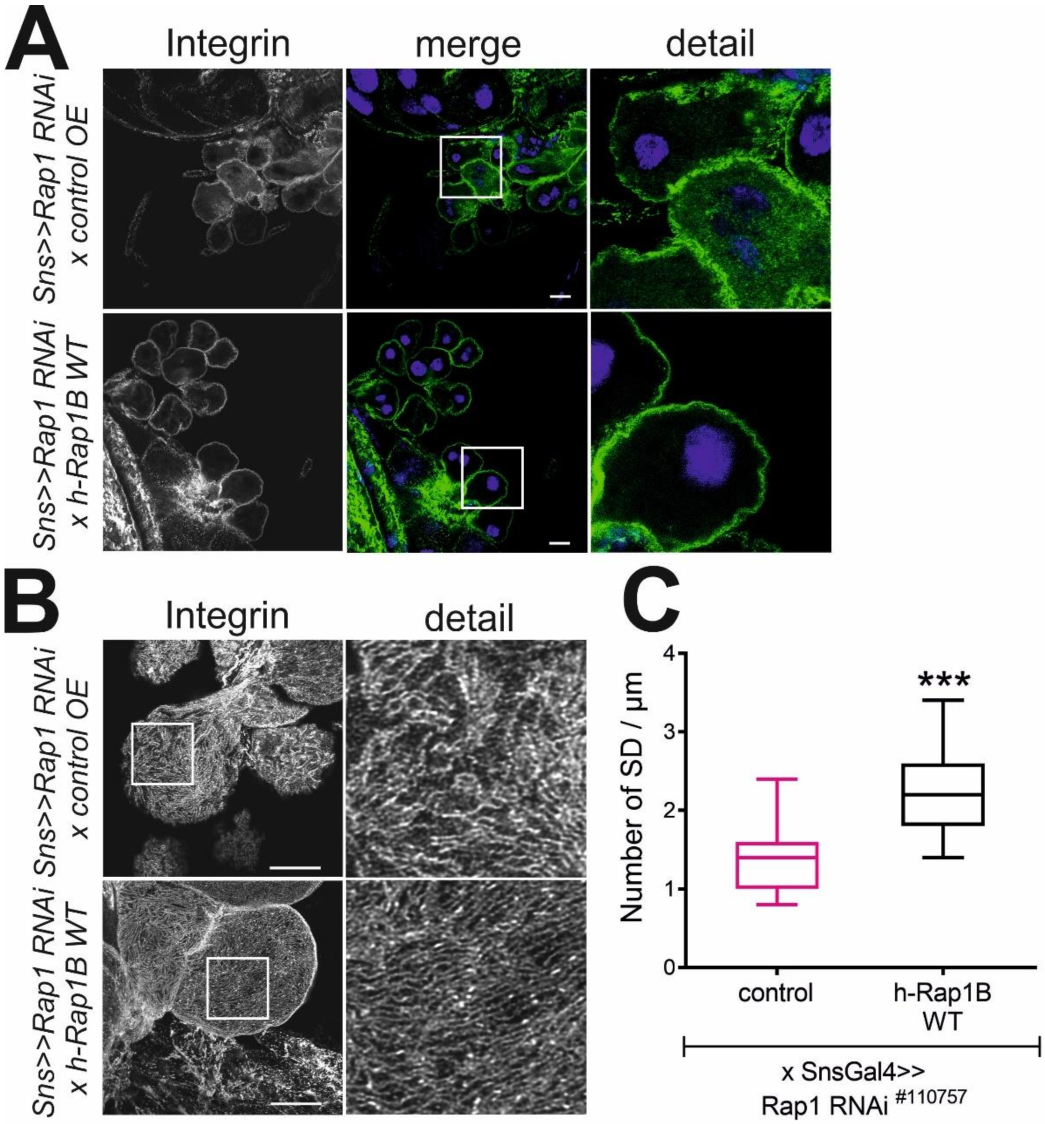

Supplementary Figure 1. Human Rap1B can rescue *rap1* loss of function-induced mislocalization of Integrin β. Immunofluorescence analysis of tangential sections (A) or surface sections (B) of *rap1* knockdown nephrocytes (*sns>>Rap1 RNAi*) with a genetic element for overexpression of human wild-type Rap1 (*h-Rap1B WT*) or an element for overexpression of a control (*control OE*) are shown. Knockdown of *rap1* (#110757) in nephrocytes was accomplished by employing *sns-GAL4*. Wandering third instar larvae were dissected and immunofluorescence analysis was performed with antibodies specific for Integrin β. Merged images and higher magnifications of the marked area (detail) are shown. Scale bars in (A) and (B): 10 µm. n=3. (C) Statistical evaluation of the number of slit diaphragms (SD) per 5 µm nephrocyte surface area of the genotypes shown in (A) and (B) depicted as a box plot and minimum and maximum. The control is marked in pink. *** $p < .001$.

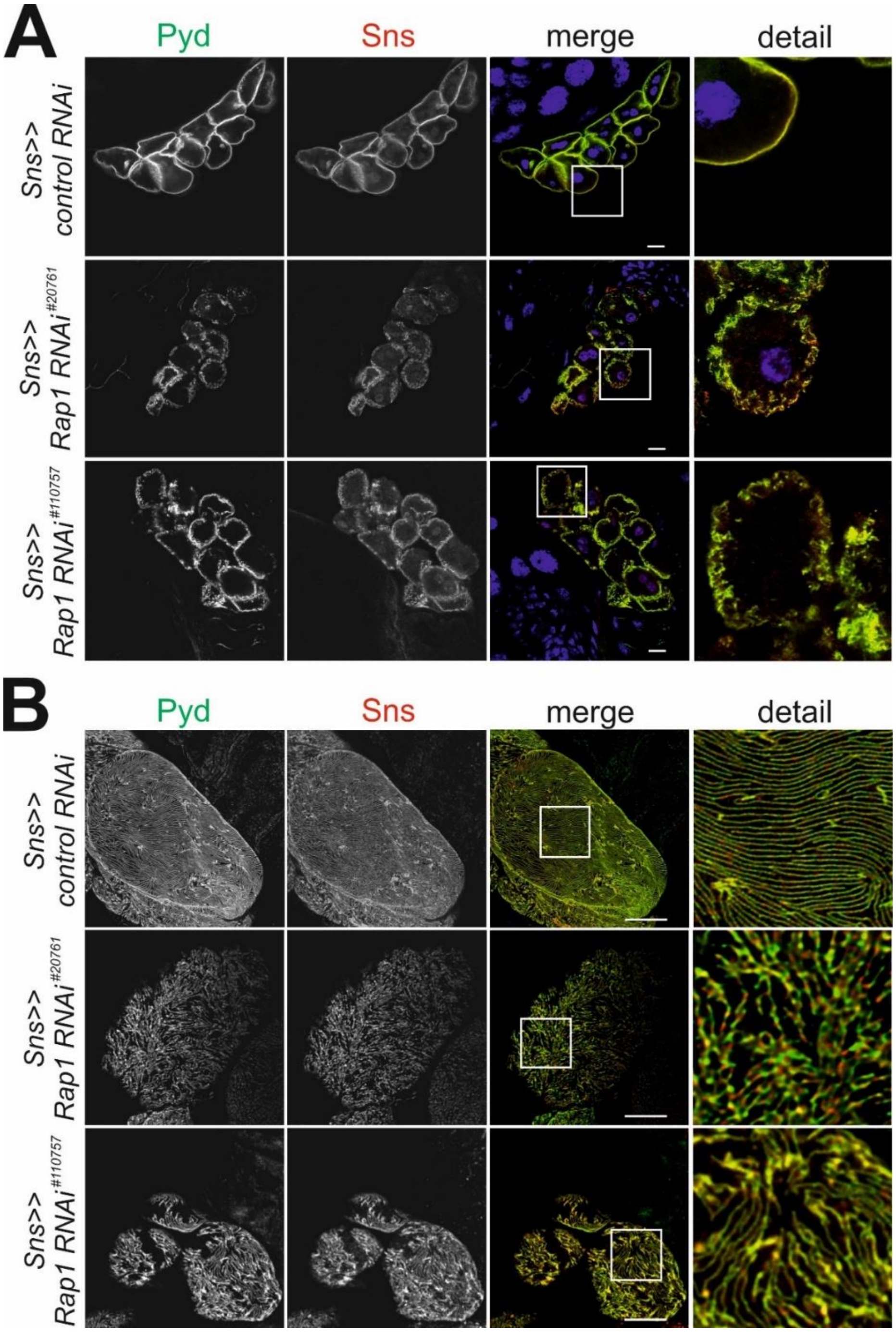

Supplementary Figure 2. Knockdown of *rap1* results in mislocalization of the slit-diaphragm proteins Pyd and Sns. (A) Immunofluorescence analysis of tangential (A) or surface sections (B) of *control* (*sns>>control RNAi*) or *rap1* knockdown nephrocytes (*sns-Gal4>>rap1 RNAi*) are shown. Knockdown in nephrocytes was accomplished by employing *sns-GAL4* and two different RNAi hairpins were employed (#20761 and #110757). Nephrocytes of wandering third instar larvae were dissected and immunofluorescence analysis was performed with antibodies specific for Zonula-occludens 1 (ZO-1, ortholog in Dm Pyd) and the Drosophila ortholog of Nephrin – Sticks and stones (Sns). Merged images and higher magnifications of the marked area (detail) are shown. Scale bars in (A) and (B): 10 µm. n=3.

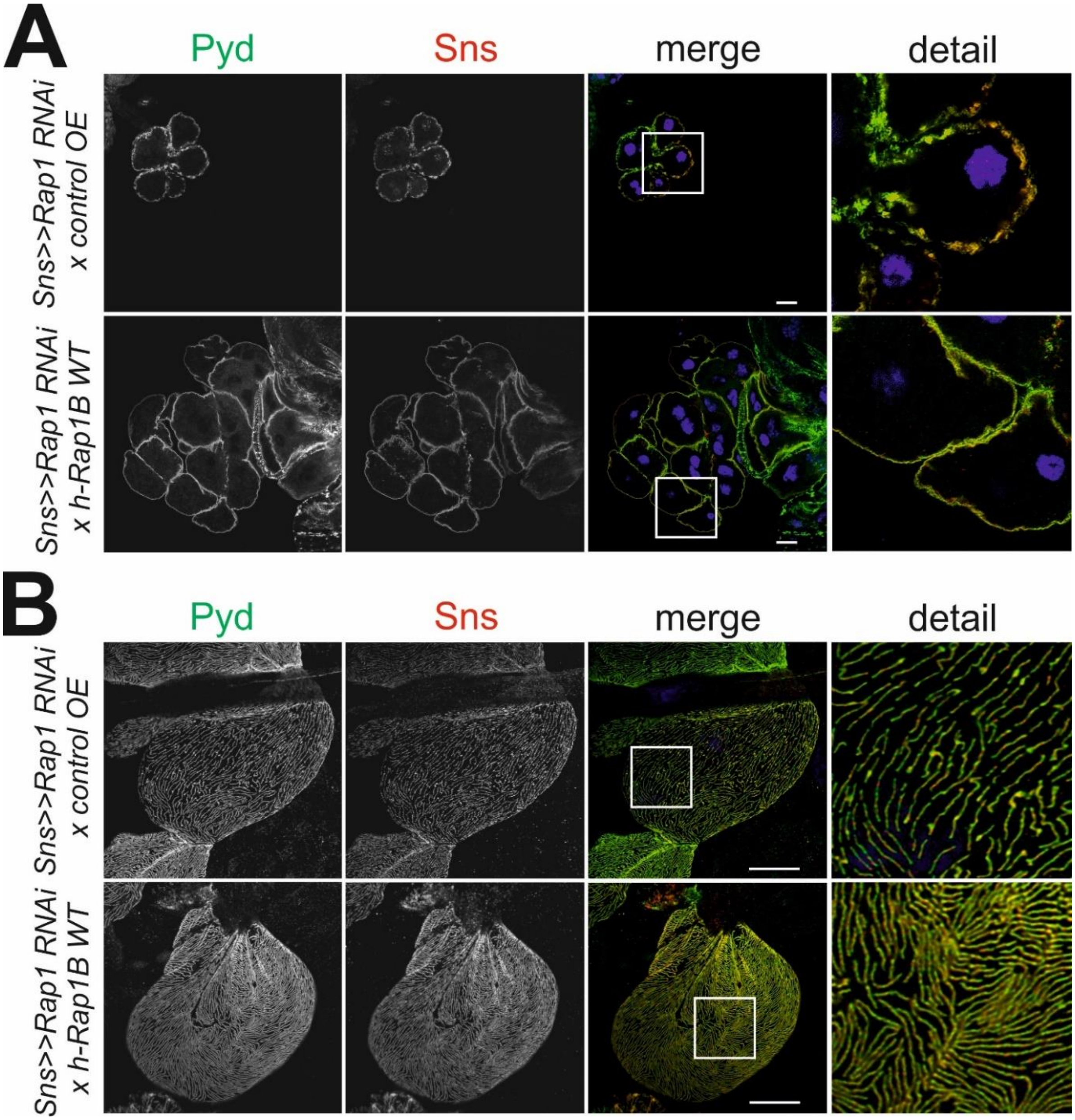

Supplementary Figure 3. Human Rap1B can rescue *rap1* loss of function-induced mislocalization of Pyd and Sns. Immunofluorescence analysis of tangential (A) or surface sections (B) of *rap1* knockdown nephrocytes (*sns>>rap1RNAi)* with a genetic element for overexpression of human wild-type Rap1B (*h-Rap1B WT*) or a control transgene (*control OE*) are shown. Knockdown of *rap1* (#20761) in nephrocytes was accomplished by employing *sns-GAL4*. Wandering third instar larvae were dissected and immunofluorescence analysis was performed with antibodies specific for Pyd and Sns. Merged images and higher magnifications of the marked area (detail) are shown. Scale bars in (A) and (B): 10 µm. n=3.

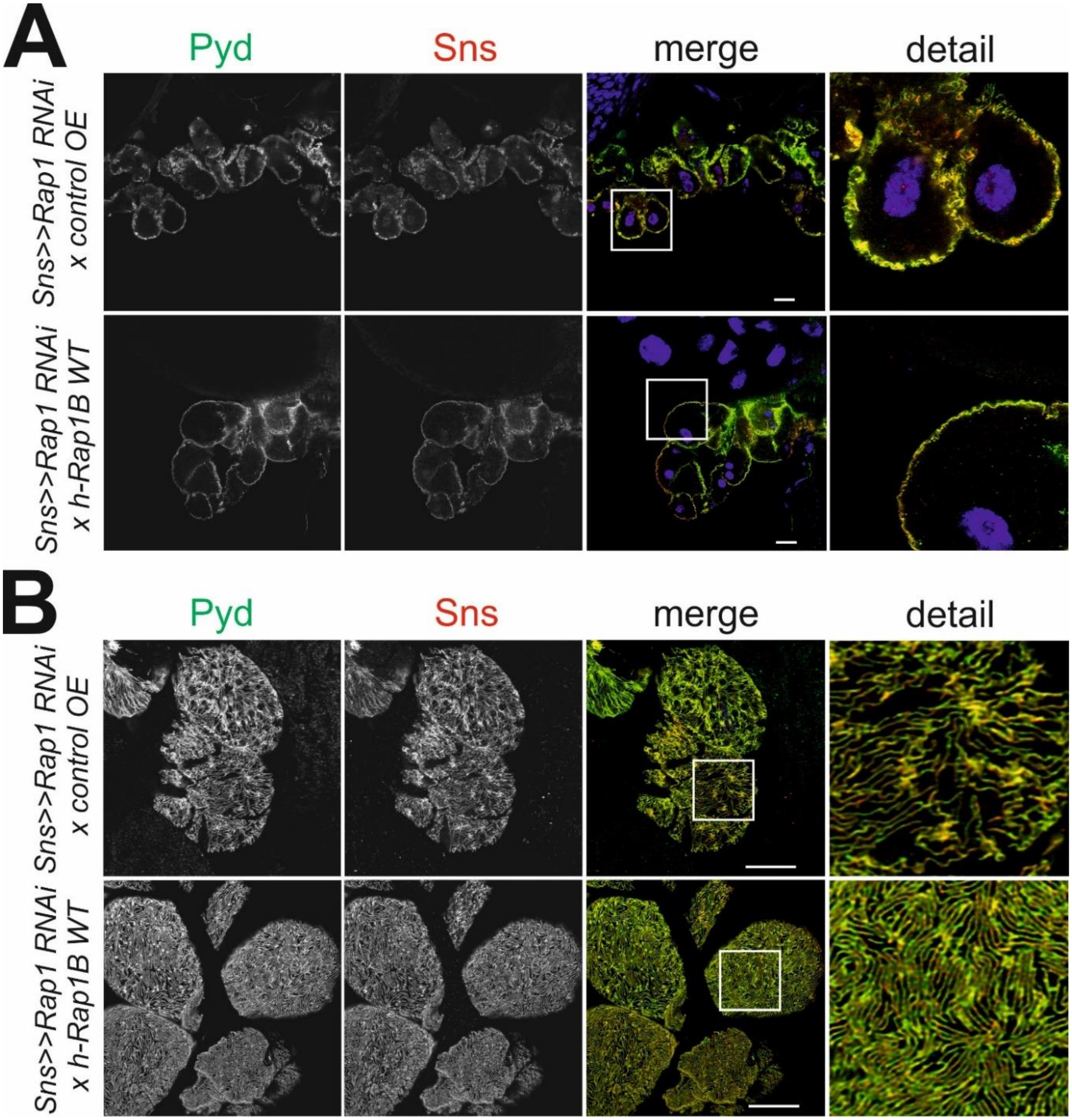

Supplementary Figure 4. Human Rap1B can rescue *rap1* loss of function-induced mislocalization of Pyd and Sns (additional information). Immunofluorescence analysis of tangential (A) or surface sections (B) of *rap1* knockdown nephrocytes (*sns>>rap1RNAi*) with a genetic element for overexpression of wild-type human Rap1B (*h-Rap1B WT*) or a control transgene (*control OE*) are shown. Knockdown of *rap1* (#110757) in nephrocytes was accomplished by employing *sns-GAL4*. Wandering third instar larvae were dissected and immunofluorescence analysis was performed with antibodies specific for Pyd and Sns. Merged images and higher magnifications of the marked area (detail) are shown. Scale bars in (A) and (B): 10 µm. n=3.

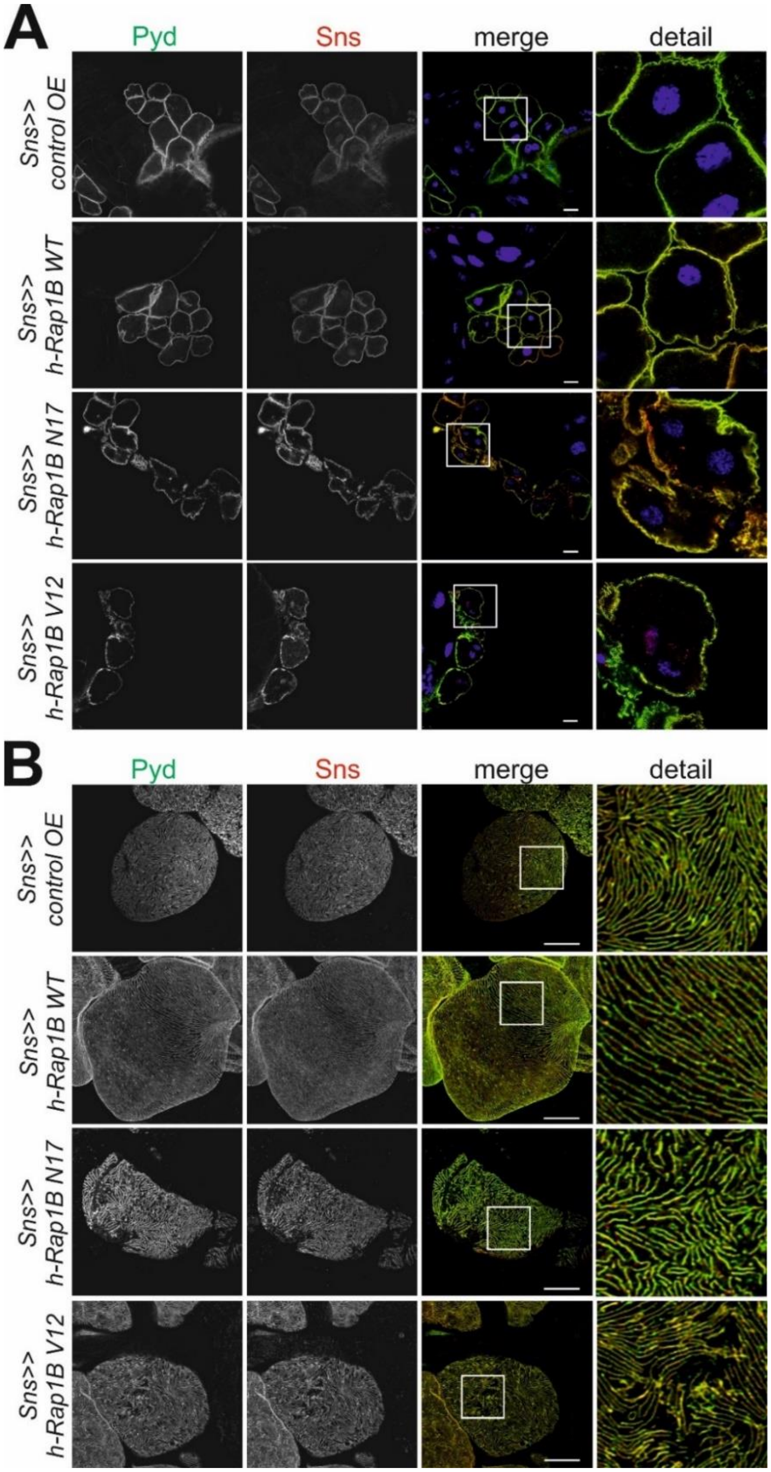

Supplementary Figure 5. Misexpression of human Rap1 results in altered targeting of the slit-diaphragm proteins Pyd and Sns. Immunofluorescence analysis of tangential (A) or surface sections (B) of control nephrocytes (*sns>>control OE*), nephrocytes with overexpression of human wild-type Rap1B (*sns>>h-Rap1B WT*), of human dominant negative Rap1B (*sns>>h-Rap1B N17*), or of human constitutively active Rap1B (*sns>>h-Rap1B V12*). Knockdown in nephrocytes was accomplished by employing *sns-GAL4*. Wandering third instar larvae were dissected and immunofluorescence analysis was performed with antibody specific for Pyd and Sns. Merged images and higher magnifications of the marked area (detail) are shown. Scale bars in (A) and (B): 10 µm. n=3.

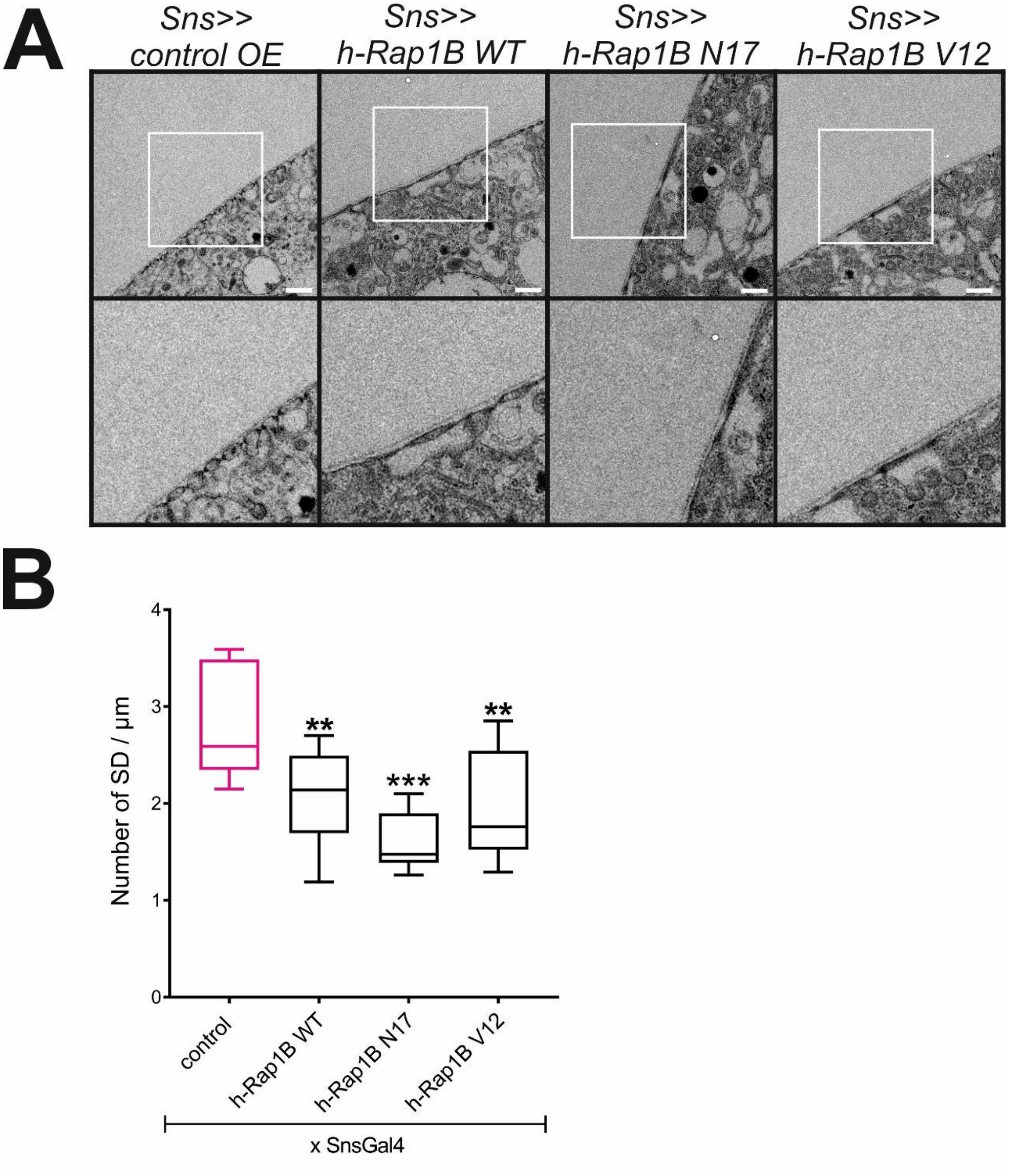

Supplementary Figure 6. Imbalance of human Rap1 activity leads to loss of nephrocyte diaphragms and lacunae. (A) TEM analysis of prepared control nephrocytes (*sns>>control OE*), nephrocytes with overexpression of human wildtype Rap1B (*sns>>h-Rap1B WT*), of human dominant negative Rap1B (*sns>>h-Rap1B N17*), or of human constitutively active Rap1B (*sns>>h-Rap1B V12*). Knockdown in nephrocytes was accomplished by employing *sns-GAL4*. White arrows exemplarily indicate slit-diaphragms. Scale bars (A) 500 nm, 2x zoom compared to the upper panel. n=3. (B) Statistical evaluation of the number of slit diaphragms (SD) per µm basement membrane of the genotypes shown in (A) depicted as a box plot. The control is marked in pink. ** $p < .01$, *** $p < .001$.

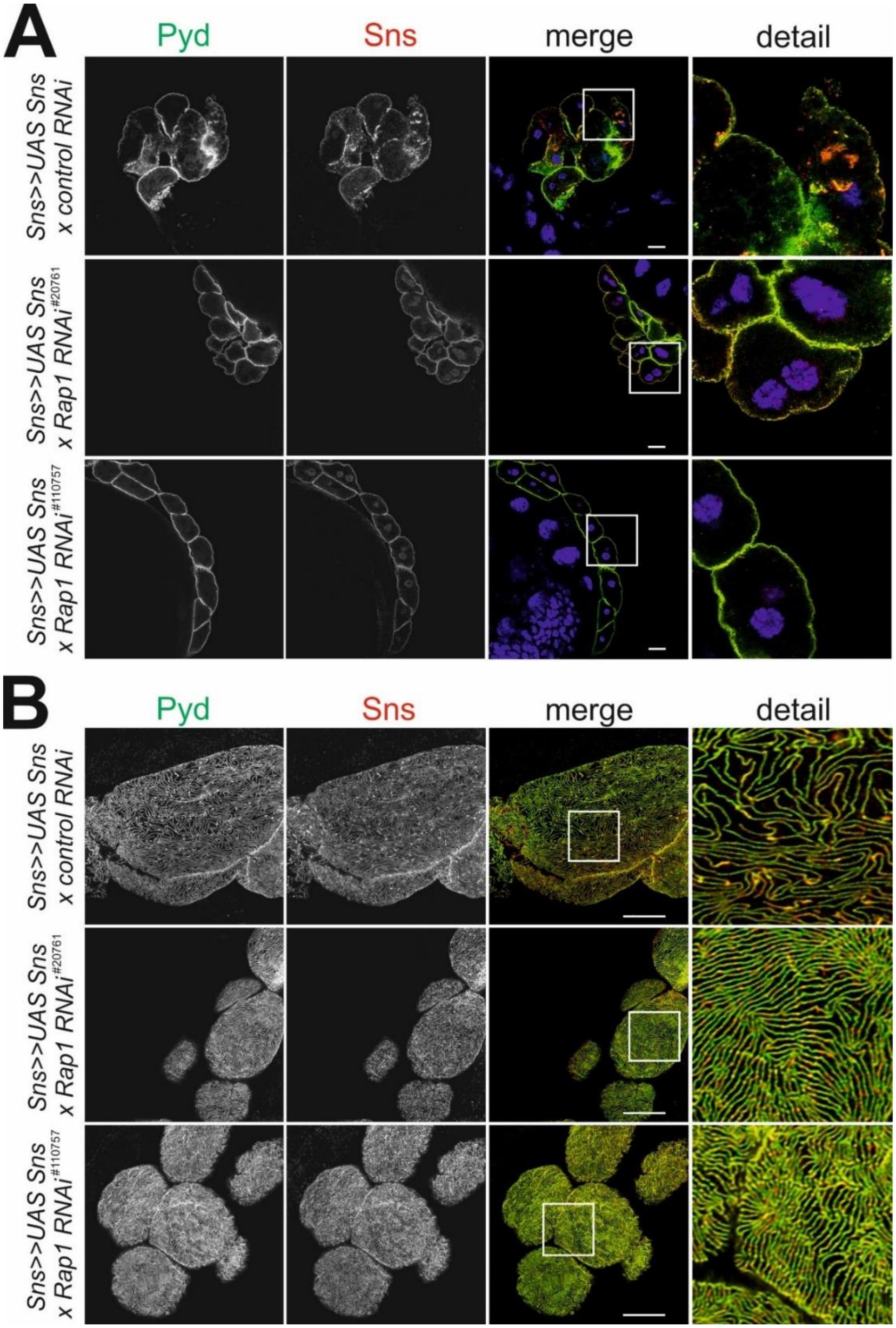

Supplementary Figure 7. Rap1 functions down-stream of Nephrin for slit-diaphragm integrity. Immunofluorescence analysis of tangential (A) or surface sections (B) of nephrocytes overexpressing *sns* (*sns>>UAS Sns x control RNAi*) or nephrocytes with overexpression of *sns* and knockdown of *d-rap1* (*sns>>UAS Sns x Rap1 RNAi*^{20761} *or sns>>UAS Sns x Rap1 RNAi*^{110757}). Knockdown in nephrocytes was accomplished by employing *sns-GAL4*. Wandering third instar larvae were dissected and immunofluorescence analysis was performed with antibodies specific for Pyd and Sns. Merged images and higher magnifications of the marked area (detail) are shown. Scale bars in (A) and (B): 10 µm. n=3.

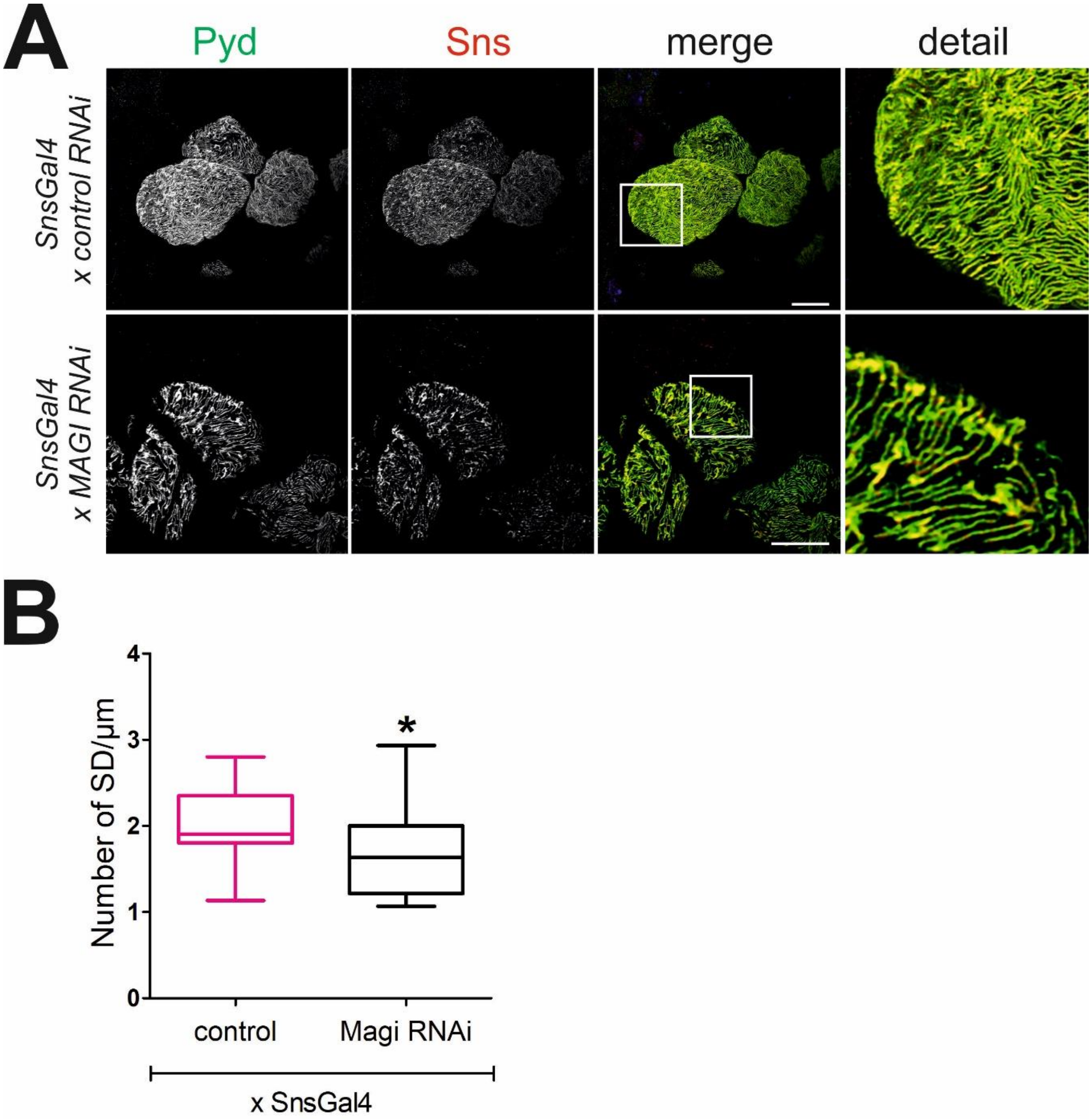

Supplementary Figure 8. Down-regulation of *magi* results in a reduction of slit-diaphragms in nephrocytes. Surface sections (A) of nephrocytes with a knockdown of *magi* or control *RNAi* element are shown (*sns>>MAGI RNAi or sns>> control RNAi*). Knockdown in nephrocytes was accomplished by employing *sns-GAL4*. Wandering third instar larvae were dissected and immunofluorescence analysis was performed with antibodies specific for Pyd (green) and Sns (red). Merged images and higher magnifications of the marked area (detail) are shown. Scale bars: 10 µm. n=3. (B) Statistical evaluation of the number of slit diaphragms (SD) per 5 µm nephrocyte surface area of the genotypes shown in (A) depicted as a box plot and minimum and maximum are shown. The control is marked in pink. * $p < .01$.
